# A bench stable formal Cu(iii) *N*-heterocyclic carbene accessible from simple copper(ii) acetate†
†All authors have given approval to the final version of the manuscript.
‡
‡Electronic supplementary information (ESI) available: Detailed experimental instructions, ^18^O-labelling procedures, all NMR data and ESI-MS data (PDF) as well as crystallographic data for **1**, **2**, **3** and **4** (CIF). CCDC 1829612–1829615. For ESI and crystallographic data in CIF or other electronic format see DOI: 10.1039/c8sc01834k


**DOI:** 10.1039/c8sc01834k

**Published:** 2018-09-14

**Authors:** Zohreh S. Ghavami, Markus R. Anneser, Felix Kaiser, Philipp J. Altmann, Benjamin J. Hofmann, Jonas F. Schlagintweit, Gholamhossein Grivani, Fritz E. Kühn

**Affiliations:** a Molecular Catalysis , Catalysis Research Center and Faculty of Chemistry , Technische Universität München , Lichtenbergstrasse 4 , D-85747 Garching bei München , Germany . Email: fritz.kuehn@ch.tum.de ; Fax: +49 89 289 13247 ; Tel: +49 89 289 13096; b School of Chemistry , Damghan University , Damghan 36715-364 , Iran

## Abstract

For years, Cu(iii)NHCs have been proposed as active intermediates in Cu(i)NHC catalyzed reactions, yielding the desired products by reductive elimination, but until today, no one has ever reported the characterisation of such a compound. When working on the synthesis of biomimetic transition metal (NHC) complexes and their application in homogeneous catalysis, we recently found a highly unusual reactivity for Cu(ii) acetate in the presence of a particular cyclic tetra(NHC) ligand. Therein, the formation of the first stable CuNHC compound, displaying Cu in the formal oxidation state +III, by simple disproportionation of Cu(ii) acetate in dimethyl sulfoxide (DMSO) was observed. At elevated temperatures selective mono-oxidation of the NHC ligand occurs, even under anaerobic conditions. Acetate was identified as the origin of the oxygen atom by ^18^O-labelling experiments. The remarkably high stability of the title compound was furthermore proven electrochemically by cyclic voltammetry. An in-depth investigation of its reactivity revealed the involvement of four additional compounds. Three of them could be isolated and characterised by ^1^H/^13^C-NMR, single crystal XRD, mass spectrometry and elemental analysis. The fourth, a Cu(i)NHC intermediate, formed by formal reductive elimination from the Cu(NHC)^3+^ compound, was characterised *in situ* by ^1^H/^13^C-NMR and computational methods.

## Abstract 2

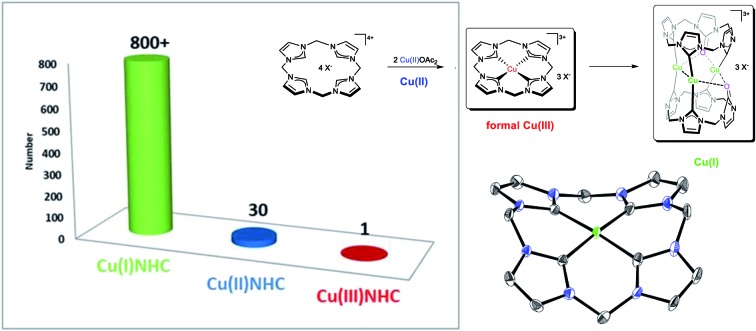
The first stable formal Cu(iii) NHC and its unusual reactivity with acetate are reported. Several products of this reaction are identified and fully characterised. It reactivity is extensively investigated and additionally explored by means of theoretical, electrochemical and isotope labelling experiments.

## Introduction

Almost a quarter of a century ago the first *N*-heterocyclic carbene (NHC) ligated Cu(i) complexes were reported by Arduengo and shortly thereafter by Raubenheimer.^1^–^3^ Until today, the overwhelming majority of currently more than 800 described Cu(i)NHCs follows the established cationic [Cu(i)(NHC)_2_]X or neutral [Cu(i)(NHC)X] motif, displaying linearly coordinated Cu(i) ions.^4^,^5^ Complexes of this type are readily accessible by various established methods such as transmetalation from the respective Ag(i)NHCs or with external bases from the imidazolium salts; furthermore, they are usually rather inert towards air in the solid state.^6^,^7^ On the other hand Cu(ii)NHCs are much more scarce than their Cu(i) analogues with around 30 reported compounds, the first of which was reported by Meyer *et al.* in 2003 featuring a tripodal NHC ligand.^8^ It is noteworthy that most Cu(ii)NHCs reported thereafter bear chelating NHC ligands with usually harder, often anionic N- or O-donor atoms, thus exploiting Cu(ii) tendency for higher coordination numbers and harder ligands.^9^–^11^ Their overall convenient preparation and good manageability, in addition to the pecuniary benefit of Cu compared to many other precious metals such as Ag, Au, Pd and Pt, make it hardly surprising that soon after the initial description, catalytic applications of Cu(i)NHCs have been reported as well.^12^,^13^ Among those were [3 + 2] cycloadditions (“click chemistry”), hydrosilylations, carbene transfer (cyclopropanation), alkyne activation and cross-coupling reactions.^7^,^14^–^19^ A number of formal Cu(iii) compounds are known in the literature, most of which bear anionic nitrogen ligands,^20^–^22^ or other strong sigma donors such as CF_3_^–^ as in Naumann's seminal [Cu(CF_3_)_4_]^–^,^23^ the likewise surprising stability of which was profoundly investigated by Snyder.^24^ However, no formal Cu(iii) complex with a neutral ligand, such as an NHC, has been isolated and reported so far. Hence, in this work the synthesis of the first stable CuNHC complex with Cu in the formal oxidation state +III and its capability to undergo reductive elimination is reported. In addition, an extensive look into the reactivity of this unique compound including a number of fully characterised follow up products as well as electrochemical characterisation of the title compound is provided.

In recent years “biomimetic” tetra(NHC) ligands were investigated, structurally reminiscent of tetrapyrrole macrocycles, which are frequently found in biological systems.^25^–^27^.

**Chart 1:**
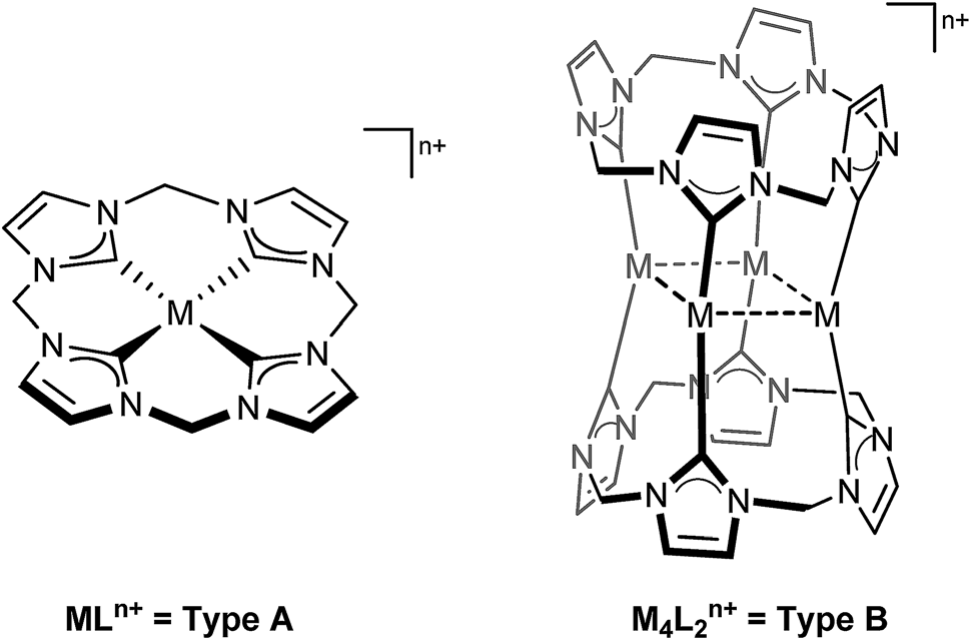
Coordination modes known for transition metal complexes of tetra(NHC) ligand L (M = Fe, Ni, Pd, Pt, Ag, Au; *n* = 2–4).^26^,^27^

Strongly dependent on the metal(s) coordinated, this type of ligand can support two distinct coordination patterns (Chart 1): A square planar coordination (**A**) is found for metals such as Fe, Ni, Pd or Pt, similar to its biological inspiration. However, being more flexible by exhibiting freely rotatable methylene moieties, instead of more rigid CH bridges, it can also form molecular box-like structures (**B**), unlike its biological counterparts.^28^

This type of coordination is strongly favoured for the coinage metals in their lower oxidation states [Ag(i), Au(i)]. Hence, in this work, the reactivity of their lighter homologue Cu, which can exhibit both of the aforementioned coordination modes (**A**, **B**), depending solely on its formal oxidation state (III, I), is investigated. Furthermore, the formation of an oxidised NHC-ligand is described, originating from a nucleophilic attack of acetate, which is unequivocally identified as the source of oxygen atom (*vide infra*) by means of ^18^O-labelling experiments. This finding is in contrast to the small number of examples of the oxidation of metal bound NHCs and further illustrates the unique properties of the title compound; in all other cases, including other Cu NHCs, molecular oxygen has been claimed to be the oxygen source.^29^–^32^

## Results and discussion

**Scheme 1:**
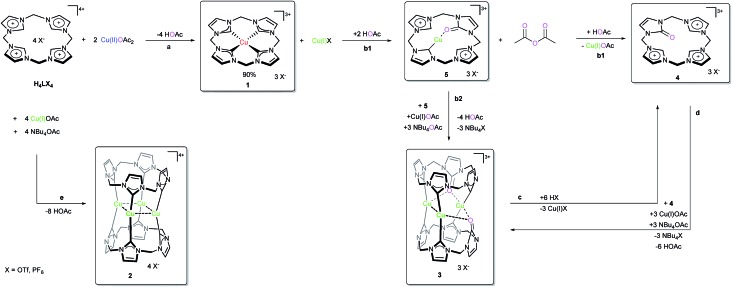
Reaction conditions and solvents ((a) 3 h, 40 °C, DMSO; (b1) 5 min, 80 °C, MeCN; b2: 10 min, 100 °C, NBu_4_OAc, DMSO or MeCN; (c) **1**, 80 °C, HX, MeCN; (d) **1**, 25 °C, Cu(i)OAc, NBu_4_OAc, DMSO; (e) 30 min, 100 °C, Cu(i)OAc, NBu_4_OAc, DMSO).

The surprisingly straightforward preparation of the Cu tetra(NHC) complex (**1**) of Type A is easily achieved by stirring Cu(ii) acetate and the ligand precursor H_4_LX_4_ (X = OTf, PF_6_) in dimethyl sulfoxide (DMSO) at room temperature. Despite being stable as a solid in air, compound **1** shows a rich reactivity under various conditions. Several defined Cu(i)NHC species (**2**, **3**, **5**) are observed, resulting from its unique reactivity with acetate. Among those, the Cu_4_L_2_ compound **2** (Scheme 1) displays the same structural motif as the related heavier coinage metal analogues (Type B).^26^,^27^ In the following sections, first the synthesis and structure of compounds **1**, **4** and **5** are discussed (for a detailed discussion of **2** and **3** see the ESI‡), consecutively establishing their chemical relationship and finally concluding with some mechanistic considerations (*vide infra*).

### Preparation and characterisation of compound **1**

The preparation of **1** is remarkably straightforward and requires neither dried solvents nor an inert gas atmosphere. Under optimised conditions, **1** is obtained in very good yields of about 90% from H_4_LX_4_ and Cu(ii) acetate at 40 °C (eqn (1)) in a disproportionation reaction. Both purity and yield of **1** decrease about 10% under exclusion of oxygen due to the competitive formation of Cu(i) complex **2** from the Cu(i)X byproduct. Under aerobic conditions, the side product is reoxidised and thus the side reaction becomes inhibited (*cf.* preparation of **2** in the ESI‡).
1

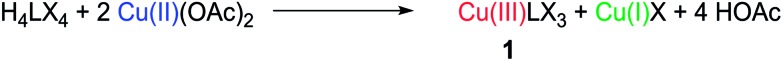




**Fig. 1:**
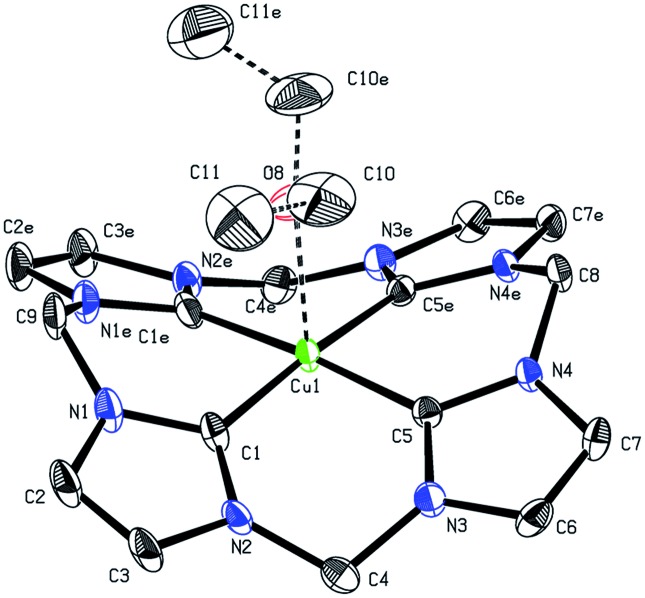
X-ray structure of the cationic fragment of compound **1**. Hydrogen atoms and counterions are omitted for clarity. Thermal ellipsoids are shown at 50% probability level. Selected bond lengths (Å) and angles (deg): Cu1–C1 1.883(5), Cu1–C5 1.879(5), Cu1–O1 2.5(2) Å, Cu1–O1A 2.3(2) Å , C5–Cu1–C5′ 90.3(3), C1–Cu1–C1′ 90.5(3), C1–Cu1–C5 89.3(2).

Since no crystal data for other formal Cu(iii)NHCs are available, **1** can only be compared with reported Cu(i)- and Cu(ii)NHC compounds. The Cu center (Fig. 1) depicts almost perfect square planar coordination (angular sum of 359.4°) and connects to a weakly coordinated O-bound, axial Et_2_O molecule (Cu–O: 2.3–2.5 Å (variance due to disorder), [Cu(ii)DMSO_4_]^2+^: 1.94 Å).^22^,^33^ The tetra(NHC) ligand itself shows a distorted saddle shape. The Cu–C distances average to 1.88 Å, which is slightly shorter than for Cu(i) (1.90 to 1.95 Å) and Cu(ii)–NHC bonds (1.89 to 1.99 Å). With regard to NMR spectroscopy a number of interesting observations can be made for complex **1**. The ^1^H-NMR spectrum in MeCN-*d*_3_ shows only one sharp singlet at 7.84 (CH) and one broad peak at 6.47 ppm at r.t. (DMSO-*d*_6_: 8.16 and 6.82, 6.43 ppm). At lower temperatures signal splitting of the peak at 6.47 ppm (CH_2_) into two doublets at 6.67 and 6.17 ppm is observed (see ESI 5.5‡). This indicates that free inversion of the ligand is frozen on an NMR timescale.^26^,^34^ Remarkably, the Cu–C resonance is strongly highfield shifted in the ^13^C-NMR (149.4 ppm).

Compared to Cu(i)–C resonances, which usually appear very close to 180 ppm and its Fe/Ni analogues (189.3/166.5 ppm) the signal of **1** is strongly up-field shifted. It is close to the chemical shifts of Au(iii)–C signals in similar Au(iii)-tetra(NHC) complexes, commonly observed between 147 and 145 ppm.^35^ This might indicate a close relationship between the electronic structure of **1** and those relatively widespread compounds.^35^,^36^ The backbone resonance at 123.2 ppm and the CH_2_ bridges at 62.4 ppm are also very close to those of Au(iii)-tetra(NHC) complexes at about 124 and 63 ppm, respectively. The elemental analysis of isolated **1** is in excellent accordance with the calculated composition derived from SC-XRD. The same applies for the ESI-MS data collected (see the Experimental). For a detailed discussion of the structures and properties of compounds **2** and **3** see the ESI.‡

### Preparation and characterisation of compound **4**

The preparation of **4** can either be achieved by the protolysis of **3** (see ESI 2.1,‡ Scheme 1 path c), or by conducting a modified protocol for the synthesis of **2** (see ESI, S-II‡) in acetonitrile and without an external base (eqn (2)).
2

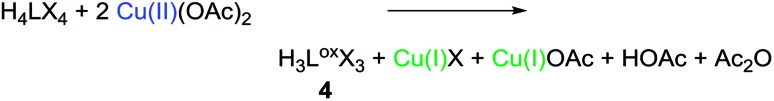




**Fig. 2:**
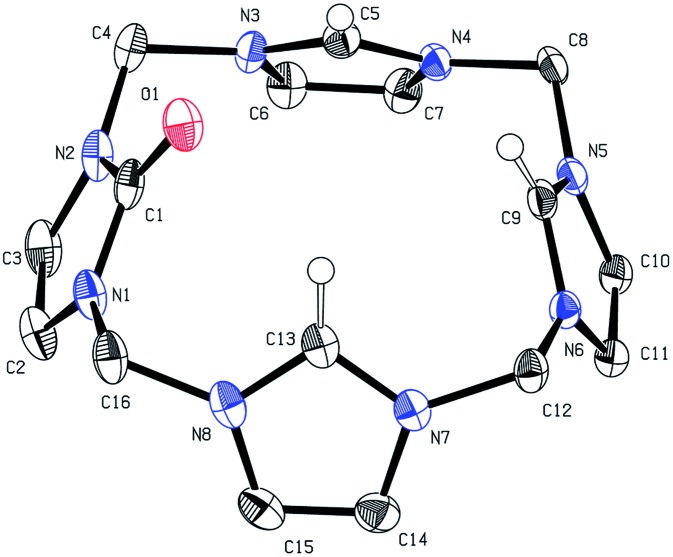
X-ray structure of the cationic fragment of compound **4**. Hydrogen atoms – except those able to from hydrogen bonds with the oxygen atom – solvent molecules and counterions are omitted for clarity. Thermal ellipsoids are shown at 50% probability level. Selected bond lengths (Å) and angles (deg): C1–O1: 1.230(3), C1–N1: 1.375(4); C1–N2: 1.376(4), C5–N3: 1.324(3); C5–N4: 1.330(3), C9–N5: 1.329(3); C9–N6: 1.327(3), C13–N7: 1.332(4); C13–N8: 1.326(3), C2–C3: 1.333(4); O1–C1–N2: 127.6(3), O1–C1–N3: 127.8(3).

Under these reaction conditions no significant amounts of **3** are formed and **4** can be isolated as a white powder in good yields (*ca.* 75%). The X-ray crystal structure of **4** is depicted below (Fig. 2). All imidazolium hydrogen atoms H5, H9 and H13 point in the same direction and are oriented towards the negatively polarised oxygen atom.^37^ The oxygen–hydrogen distances of the neighbouring H-atoms are between 2.6 and 2.7 Å, which is a medium strong interaction, according to Jeffrey.^38^ The hydrogen H9 shows a distance of around 3.8 Å, which is significantly longer and classifies this H–O contact only as a “weak, electrostatic” interaction.^37^ The bridging CH_2_-groups only display two distinct singlets (6.53, 5.88 ppm in CD_3_CN), indicating free rotation of the imidazolium moieties at r.t. Naturally, compound **4** can be converted into **3**, simply by stoichiometric addition of Cu(i) acetate and NBu_4_OAc (2 : 3 : 3; eqn (3)).
3

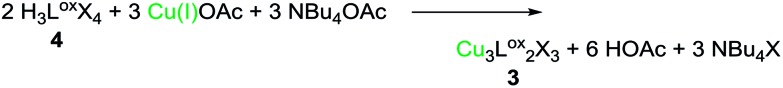




### Characterisation of intermediate compound **5**

If the synthesis of **4** (eqn (2), Scheme 1) is carried out under strictly anaerobic conditions, another compound **5** can be observed with the help of *in situ* NMR experiments. Despite many efforts it was not possible to isolate compound **5**. Interestingly, this intermediate is exclusively observed in MeCN, not in DMSO, for various temperatures and ligand to acetate ratios. An explanation for this finding might be the much higher stability of the Cu(i) DMSO complex compared to the Cu(i) MeCN complex, thus DMSO prevents the formation of the relatively labile compound **5**.^39^ Its formation could be studied preferably by heating a solution of H_4_LX_4_ and Cu(ii) acetate (1 : 2) in MeCN for 10 min at 80 °C in a sealed NMR-tube, yielding approximately a 1 : 1 mixture of **4** and **5**.

**Fig. 3:**
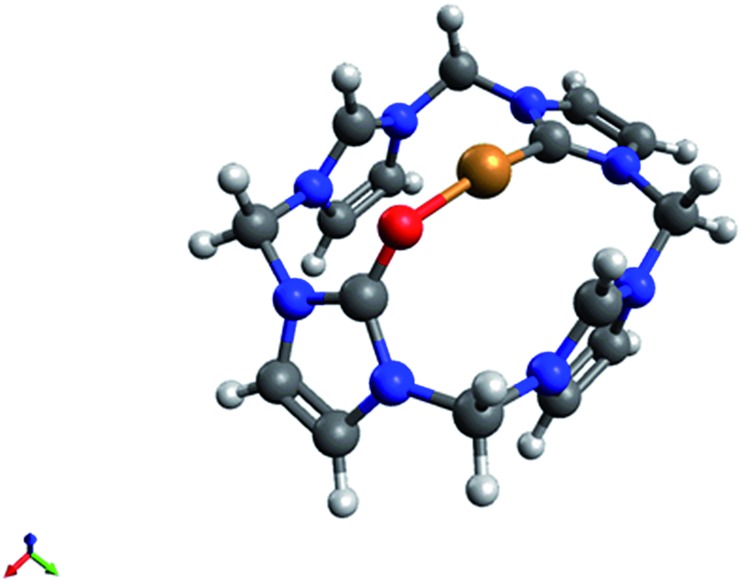
DFT-derived structure for the proposed cationic fragment [CuH_2_Lox]^3+^ of **5** (B3LYP, def2-TZVPD). Selected calculated bond lengths (Å) and angles (°): Cu1–C1: 1.894, Cu1–C2: 2.758, Cu1–C4: 2.758, Cu1–O1: 1.886, C3–O1: 1.260, O1–H1: 3.083, O1–H2: 3.083, C1–Cu1–O1: 168.9, C3–O1–Cu1: 121.5.

This solution can be stored at r.t. for several days, however exposure to air resulted in an immediate decomposition of **5**, only leaving **4** and a blue solution (presumably Cu(ii)) behind. Compound **5** was studied by *in situ* 1D and 2D NMR spectroscopy and DFT calculations. Its ^1^H- and ^13^C-NMR spectra are closely related to that of **3**, hence being formed by a formal H^+^ to Cu^+^ exchange at the C9-carbon atom (Fig. 3). The signal splitting of the bridging CH_2_ groups from two singlets (6.74, 6.04 ppm) in **4**, into four doublets (6.52, 6.11, 6.06, 5.60 ppm) in **5** clearly shows the loss of rotational freedom, thus pointing to a coordination of the opposite oxygen atom. The observation of a single, relatively low field Cu–C_carbene_ signal at 187.9 ppm, and the disappearance of the corresponding proton signal of **4** at 9.25 ppm also conclusively support the proposed structure. The calculated C–O distance in **5** is slightly longer than in **4** (1.26 Å *vs.* 1.23 Å), comparable to a similar Cu(i)NHC structure reported by Owen *et al.*^31^

The calculated Cu(i)–C_Carbene_ distance in **5** (1.89 Å) is almost identical to **2**, **3** or other Cu(i)NHC bond lengths (≈1.91 Å).

### Electrochemical properties of **1**

**Fig. 4:**
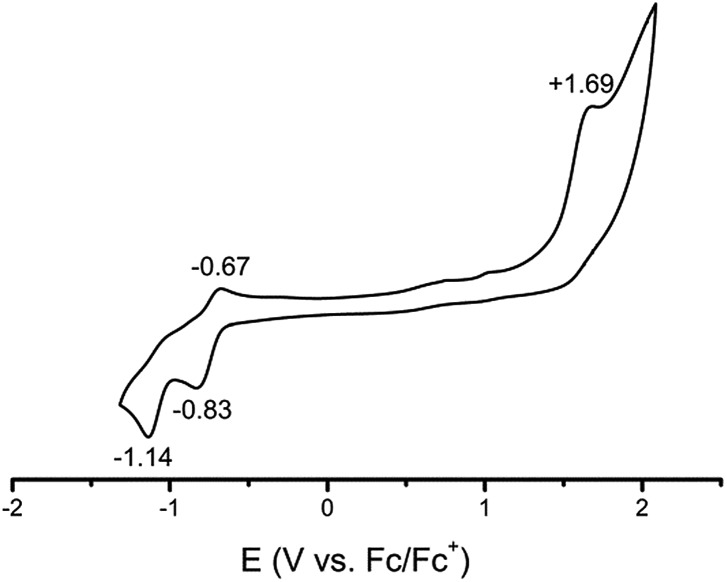
Cyclic voltammogram of **1** measured in MeCN at 20 °C (0.1 M NBu_4_PF_6_ electrolyte, scan rate 100 mV s^–1^, platinum working electrode).

To gain better experimental insight into the electronic structure and its electrochemical behaviour, **1** is subjected to cyclic voltammetry. The CV of **1** (see Fig. 4) exhibits a wide area of stability from –0.5 to +1.5 V, which coincides nicely with its observed stability under ambient conditions.

Two one electron reductions (–0.83 and –1.14 V) and one oxidation event (+1.69 V) are observed. The reduction peak at –0.75 V seems to be partially reversible, suggesting the formation of a metastable CuL^2+^ species. Further reduction (–1.14 V) as well as oxidation (+1.69 V) is irreversible. The oxidation event is attributed to the formation of a CuL^4+^ species, which consecutively decomposes. Unlike the homologous iron complex, compound **1** is highly sensitive to changes in the overall oxidation state.^25^,^40^.

This behaviour may be ascribed to the different coordination modes of later coinage metals, ranging from linear in the oxidation state +I to square planar in +III as also presented here for the formal Cu(iii) species **1** and Cu(i) species **2**. By changing the oxidation state, a recoordination becomes necessary and the complex decomposes.

### Reactivity and proposed mechanism

The discrepancy between the formal oxidation states of the Cu ions in the metal precursor (Cu(ii)) and the final complexes (Cu(iii), Cu(i)) is apparent (Scheme 1). The simplest explanation for the oxidation of Cu would be oxygen, as already described for other Cu(NHC)s.^31^,^32^ However, for the formation of title compound **1**, this can be excluded, since it is also formed in good yields in the absence of oxygen. The appearance of Cu(i) complex **2** as a by-product under anaerobic conditions, along with similar reports for comparable non-NHC Cu(iii) compounds, strongly supports the proposed disproportionation of Cu(ii) acetate (formula (1)).^22^,^41^ In combination with the ^18^O-labelling experiments (*vide infra*) any direct involvement of molecular oxygen is ruled out. The formation of **1** already starts at r.t. and is completed within 3 h at 40 °C (see ESI 5.1‡). Using MeCN as the solvent, the formation of **4** together with intermediate **5** is observed using stoichiometric amounts of acetate and heating to 80 °C (Scheme 1, pathway b1 and ESI 5.2‡). If an excess of acetate is present, the smooth formation of **3** is observed, both in DMSO and MeCN (Scheme 1, pathway b2). This difference can be exploited for the targeted synthesis of **4**.

Overall, after gaining a good understanding of **1** and its related compounds **2** to **5** one more question remains open: Where does the oxygen atom in **3** and **4** originate from? Therefore, four potential sources of oxygen are identified: DMSO, residual water, residual oxygen and acetate.

Since the formation of **3** and **4** also proceeds readily in MeCN, DMSO can be excluded as the oxygen source. To unequivocally distinguish between the other possibilities, ^18^O-labeling experiments are conducted (see the ESI: 2.2 and 3‡). Working under strict exclusion of oxygen and its presence having no significant influence on the formation of **3** or **4**, molecular oxygen can also be ruled out as the oxygen source. However, since oxygen was reported to be the oxidant for a number of other oxidised NHCs in the literature, this possibility is finally excluded by synthesizing **3** in an ^18^O_2_ atmosphere (details in ESI 2.2‡).^31^,^32^ Expectedly, no significant amount of ^18^O (1.6 ± 1.2%) is incorporated in **3** in this case. Addition of ^18^O-labelled water to the reaction mixture results in no significant increase of the ^18^O content in **3** (0.4 ± 1.4%), either. In contrast, when the reaction is conducted with ^18^O-labelled Cu(ii) acetate (≈50% incorporation of ^18^O), significant ^18^O enrichment is found in **3** (28 ± 1.8%). This matches the calculated yields according to the experimental procedures, in which additional 2.2 equivalents of unlabelled acetate (with respect to the ligand) are added. When the formation of **4** is also conducted with ^18^O-labelled Cu(ii) acetate (without addition of unlabelled NBu_4_OAc), an almost doubled ^18^O enrichment of 53 ± 0.7% is determined. By carefully monitoring the reaction progress with the help of ^1^H NMR, the fate of the resulting acetyl fragment after O-transfer becomes clear: acetic anhydride is identified as a stoichiometric side product (2.21 ppm in DMSO). Therefore, acetate is unequivocally identified as the source of the oxygen in compounds **3** and **4**.

**Scheme 2:**
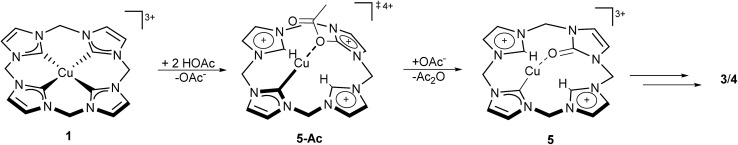
Proposed reaction mechanism for the formal oxidation of the tetra(NHC) ligand of compound **1** in the presence of acetate, leading to compounds **3** and **4**, based on ^18^O-labelling and ^1^H-NMR experiments (see the ESI‡).

These findings at hand, combined with the earlier described observation of **5** as an intermediate, a preliminary mechanism can be proposed (Scheme 2). After initial binding of acetate to the formal Cu(iii) complex, a formal intramolecular redox reaction is suggested, leading to the Cu(i) species **5-Ac**. The acetyl fragment is removed by an additional equivalent of acetate, giving acetic anhydride and **5**.

The mode of interaction between acetate and **1** is investigated *via*^1^H NMR titration experiments by stepwise addition of NBu_4_OAc to the complex. Therein, the simple two singlets of **1**, resulting from its D_4h_ symmetry, successively split into a more complex pattern, indicating symmetry breaking.

This suggests the formation of an asymmetric acetate species, which cannot be expected for a purely metal bound mechanism (ESI 5.6‡). Thus, either significant hydrogen bonding is involved in a mere metal centred acetate-**1** adduct, hindering free rotation of the apical acetate, or the formation of the adduct is ligand centred.

Either mode of interaction, as well as the intramolecular redox reaction, is highly interesting, as such a behaviour has never been observed before with comparable carbene metalates.

Extensive further experiments and DFT calculations are ongoing in our facilities to fully elucidate the underlying mechanism as well as reactivity and electronic properties of the involved compounds, especially **1**. Furthermore, the full synthetic and catalytic potential of this unprecedented reactivity shall be explored.

## Conclusions

A straightforward synthesis of the first stable Cu(NHC) compound with Cu in the formal oxidation state +III from the inexpensive und simple Cu(ii) acetate is introduced. The remarkable stability of the new compound is proven by cyclic voltammetry. Ample evidence is provided for its ability to allow the selective monooxidation of the ligand upon formal reductive elimination of Cu(i). The source of the O-atom has been unambiguously identified to be an acetate ion by the help of ^18^O-labelling experiments. Additionally, the reactivity of the [Cu(NHC)]^3+^ moiety is investigated, including a preliminary mechanism and identifying all significant products and intermediates of the reported reactions.

The presented highly stable, formal Cu(iii) compound is an excellent model system to study elusive, highly oxidised copper NHC complexes within countless, especially catalytic applications.

Its unusual reactivity might open up new applications in Cu(NHC) catalysed chemistry. Therefore, in-depth investigations such as detailed DFT calculations and synthetic examinations of Cu(NHC) **1** are currently underway to allow a closer insight into the electronic structure, the mechanism of follow-up reactions as well as the full synthetic and catalytic potential of that compound.

## Experimental

### Computational analyses

Geometry optimizations of the gas-phase structure have been performed using density functional theory (DFT) with the B3LYP^42^,^43^ functional and 6-31G+ as well as the def2-TZVPD^44^,^45^ basis set under the inclusion of the dispersion model GD3BJ^46^ to investigate the influence of different basis sets on the bonding properties (only the cationic part has been considered). The initial structure of 5 was taken from the experimentally obtained X-ray structure of 4; Cu(i) was added and the resulting geometry preoptimized using force field methods. Subsequent frequency analysis confirmed the structures obtained by DFT calculations being local minimums on the respective potential energy surfaces. All calculations have been carried out with the Gaussian16 software.^47^

### General procedures and analytical methods

Unless otherwise stated, all manipulations were performed under an argon atmosphere using standard Schlenk and glovebox techniques. Solvents were obtained water- and oxygen-free from an Mbraun solvent purification system. Acetonitrile-*d*_3_ was refluxed over phosphorus pentoxide and distilled prior to use. Dimethylsulfoxide-*d*_6_ was refluxed over calcium hydride and distilled prior to use. All solvents were stored over molecular sieves 3 Å. The tetraimidazolium salts (H_4_L-PF_6_/H_4_L-OTf) were synthesised according to known literature procedures.^27^ All other reagents were purchased from commercial suppliers and used without further purification. NMR spectra were recorded on a Bruker Avance DPX 400 (^1^H NMR, 400.13 MHz; ^13^C NMR, 100.53 MHz; ^19^F NMR, 376.49 MHz), and chemical shifts are reported relative to the residual signal of the deuterated solvent.^48^ LIFDI HRMS measurements were conducted on a liquid injection field desorption ionisation cell obtained from Linden CMS GmbH, Leeste, Germany. Elemental analyses (C/H/N) were performed at the microanalytical laboratory of the Technische Universität München. Electrospray ionization (ESI) mass spectrometry (MS) data were acquired on a Thermo Fisher Ultimate 3000. Single crystals of **1–4** suitable for X-ray diffraction were obtained by slow diffusion of dichloromethane into an acetonitrile/dimethyl sulfoxide solution of the respective complexes. All reactions described in the following section can also be performed with [calix[4]imidazolium][triflate], respectively.

#### [CuL](PF_6_)_3_ (**1**)

[Calix[4]imidazolium]-[hexafluorophosphate] (260.0 mg, 0.29 mmol) and Cu(ii) acetate monohydrate (120.0 mg, 0.60 mmol) were dissolved in 5 mL of technical grade DMSO in air. The blue reaction mixture was stirred at r.t. for 5 min and then stirred at 40 °C for 3 h. The clear green solution was cooled to r.t and precipitated with DCM (20 mL) forming a fine white precipitate. The clear blue supernatant solution was filtered of and the white residue was dissolved in a small amount of DMSO. This procedure was repeated twice and the white residue was consecutively washed twice with DCM. After drying under vacuum, a white powder was obtained (250.0 mg, 0.26 mmol, 90% yield). ^1^H NMR (400 MHz, DMSO-*d*_6_, 296 K): *δ* 8.16 (s, 8H, *CH*), ∼6.82/6.43 (bp, 8H, *CH*_2_) ppm. ^13^C{^1^H} NMR (101 MHz, DMSO-*d*_6_, 296 K): *δ* 150.0 (*C*_*carbene*_), 123.8 (*CH*), 63.0 (*CH*_2_) ppm. ^1^H NMR (400 MHz, MeCN-*d*_3_, 296 K): 7.84 (s, 8H, *CH*), ∼6.47 (bp, 8H, *CH*_2_) ppm. ^13^C{^1^H} NMR (101 MHz, MeCN-*d*_3_, 296 K): *δ* 150.9 (*C*_*carbene*_), 124.7 (*CH*), 64.1 (*CH*_2_) ppm. Anal. calc. for C_16_H_16_CuF_18_N_8_P_3_ + 2 DMSO: C 24.64; H 2.89; N 11.49; S 6.58; Cu 7.74. Found: C 24.70; H 2.88; N 11.42; S 6.57; Cu 7.90. ESI-MS: *m*/*z* = 402 [C_16_H_15_CuFN_8_]^+^; 527 [C_16_H_15_CuF_6_N_8_P]^+^; 672 [C_16_H_15_CuF_12_N_8_P_2_]^+^.

#### Cu_4_L_2_–PF_6_ (**2**)

[Calix[4]imidazolium][hexafluorophosphate] (500 mg, 0.55 mmol), Cu(i) acetate (141 mg, 1.16 mmol) and NBu_4_OAc (332 mg, 1.10 mmol) were dissolved in 5 mL of DMSO under an argon atmosphere. The reaction mixture was stirred at r.t. for 5 min and then heated to 100 °C for 30 min. The light brown solution was evaporated (100 °C, Schlenk vacuum), yielding a pink solid. The residue was suspended with 2 mL of MeCN resulting in a white precipitate and a pink supernatant solution. The supernatant solution was filtered off and the procedure was repeated once with the remaining white solid. To completely remove NBu_4_PF_6_ the white residue was dissolved in the minimum amount of DMSO and was precipitated with MeCN. Successively the precipitate was washed twice with diethyl ether and dried under vacuum to obtain a white powder (300 mg, 74% yield).^1^H NMR (400 MHz, DMSO-*d*_6_, 296 K): *δ* 7.71 (s, 16H, *CH*), 6.99 (d, 8H, ^2^*J*_*HH*_ = 14.6 Hz, *CH*_2_), 6.40 (d, ^2^*J*_*HH*_ = 14.6 Hz, 8H, *CH*_2_) ppm. ^13^C{^1^H} NMR (101 MHz, DMSO-*d*_6_, 296 K): *δ* 178.8 (*C*_*carbene*_), 122.1 (*CH*), 64.0(*CH*_2_) ppm. Anal. calc. for C_32_H_32_Cu_4_F_24_N_16_P_4_ + 3 DMSO: C 26.70; H 2.95; N 13.11; S 5.63. Found: C 26.27; H 2.83; N 13.21; S 5.52. LIFDI-MS: *m*/*z* = 360 [C_32_H_32_Cu_4_F_6_N_16_P + C_2_H_3_N]^3+^; ESI-MS: *m*/*z* = 383 [C_16_H_16_CuN_8_]^+^, 595 [C_16_H_19_F_12_N_8_OP_2_]^+^.

#### Cu_3_LO_2_–PF_6_ (**3**)

[Calix[4]imidazolium]-[hexafluorophosphate] (100 mg, 0.11 mmol), Cu(ii) acetate monohydrate (45.0 mg, 0.22 mmol) and NBu_4_OAc (70.0 mg, 0.23 mmol) were dissolved in 2 mL of DMSO in air. The initial blue reaction mixture was stirred at r.t. for 5 min and then heated to 100 °C for 10 min. The clear green solution was evaporated (100 °C, Schlenk vacuum) yielding a green solid. The residue was dissolved in 2 mL of MeCN and precipitated with DCM. After centrifugation a yellow solid separated from the blue supernatant solution. The supernatant solution was decanted off and the procedure was repeated twice with the remaining solid. Successively the precipitate was washed twice with DCM and dried at 70 °C in air to obtain a yellow powder (71 mg, 91% yield).^1^H NMR (400 MHz, DMSO-*d*_6_, 296 K): *δ* 7.80 (d, 2H, ^2^*J*_*HH*_ = 1.7 Hz, *CH*_*Im1a*_), 7.72 (d, 2H, ^2^*J*_*HH*_ = 1.7 Hz, *CH*_*Im2a*_), 7.69 (d, 2H, ^2^*J*_*HH*_ = 1.7 Hz, *CH*_*Im3a*_), 7.69 (d, 2H, ^2^*J*_*HH*_ = 13.3 Hz, *CH*_*2(1a)*_), 7.55 (d, 2H, ^2^*J*_*HH*_ = 1.7 Hz, *CH*_*Im1/2/3b*_), 7.53 (d, 4H, ^2^*J*_*HH*_ = 1.7 Hz, *CH*_*Im1/2/3b*_), 6.98 (d, 2H, ^2^*J*_*HH*_ = 13.5 Hz, *CH*_*2(2a)*_), 6.97 (d, 2H, ^2^*J*_*HH*_ = 2.9 Hz, *CH*_*Imoxa*_), 6.90 (d, 2H, ^2^*J*_*HH*_ = 2.9 Hz, *CH*_*Imoxb*_), 6.38 (d, 2H, ^2^*J*_*HH*_ = 13.5 Hz, *CH*_*2(2b)*_), 6.26 (d, 2H, ^2^*J*_*HH*_ = 14.2 Hz, *CH*_*2(3a)*_), 6.19 (d, 2H, ^2^*J*_*HH*_ = 13.3 Hz, *CH*_*2(1b)*_), 6.10 (d, 2H, ^2^*J*_*HH*_ = 14.2 Hz, *CH*_*2(4a)*_), 5.80 (d, 2H, ^2^*J*_*HH*_ = 13.5 Hz, *CH*_*2(3b)*_), 5.75 (d, 2H, ^2^*J*_*HH*_ = 14.2 Hz, *CH*_*2(4b)*_) ppm. ^13^C{^1^H} NMR (101 MHz, DMSO-*d*_6_, 296 K): *δ* 180.5 (*C*_*carbene1*_), 180.0 (*C*_*carbene2*_), 179.9 (*C*_*carbene3*_), 151.8 (C=O), 122.6 (*CH*_*Im3a*_), 122.0 (*CH*_*Im1a*_), 121.3(*CH*_*Im2a*_), 121.1 (*CH*_*Im1b-3b*_), 120.9 (*CH*_*Im1b-3b*_), 120.7 (*CH*_*Im1b-3b*_), 111.9(*CH*_*Imox2*_), 111.8 (*CH*_*Imox1*_), 63.8 (*CH*_*2(2)*_), 61.0 (*CH*_*2(1)*_), 56.9 (*CH*_*2(3)*_), 56.1 (*CH*_*2(4)*_) ppm. ^1^H NMR (400 MHz, MeCN-*d*_3_, 296 K): *δ* 7.79 (d, 2H, ^2^*J*_*HH*_ = 13.7 Hz, *CH*_*2(1a)*_), 7.44 (d, 2H, ^3^*J*_*HH*_ = 1.8 Hz, *CH*_*Im1a*_), 7.41 (d, 2H, ^3^*J*_*HH*_ = 1.8 Hz, *CH*_*Im2a*_), 7.35 (d, 2H, ^3^*J*_*HH*_ = 1.8 Hz, *CH*_*Im3a*_), 7.27 (d, 2H, ^3^*J*_*HH*_ = 1.8 Hz, *CH*_*Im1/2/3b*_), 7.24 (d, 4H, ^3^*J*_*HH*_ = 1.8 Hz, *CH*_*Im1/2/3b*_), 7.00 (d, 2H, ^2^*J*_*HH*_ = 14.9 Hz, *CH*_*2(2a)*_), 6.72 (d, 2H, ^2^*J*_*HH*_ = 14.9 Hz, *CH*_*Imoxa*_), 6.68 (d, 2H, ^2^*J*_*HH*_ = 14.5 Hz, *CH*_*Imoxb*_), 6.29 (d, 2H, ^2^*J*_*HH*_ = 13.5 Hz, *CH*_*2(3a)*_), 6.12 (d, 2H, ^2^*J*_*HH*_ = 14.2 Hz, *CH*_*2(2b)*_), 6.07 (d, 2H, ^2^*J*_*HH*_ = 13.3 Hz, *CH*_*2(4a)*_), 5.95 (d, 2H, ^2^*J*_*HH*_ = 14.2 Hz, *CH*_*2(1b)*_), 5.57 (d, 2H, ^2^*J*_*HH*_ = 13.5 Hz, *CH*_*2(3b)*_), 5.51 (d, 2H, ^2^*J*_*HH*_ = 14.2 Hz, *CH*_*2(4b)*_) ppm. ^13^C{^1^H} NMR (101 MHz, MeCN-*d*_3_, 296 K): *δ* 182.6 (*C*_*carbene1*_), 182.1 (*C*_*carbene2*_), 182.5 (*C*_*carbene3*_), 153.3 (C=O), 123.5 (*CH*_*Im3a*_), 122.9 (*CH*_*Im1a*_), 122.2 (*CH*_*Im2a*_), 122.1 (*CH*_*Im1b-3b*_), 122.1 (*CH*_*Im1b-3b*_), 121.8 (*CH*_*Im1b-3b*_), 113.0 (*CH*_*Imox2*_), 112.9 (*CH*_*Imox1*_), 65.4 (*CH*_*2(2)*_), 62.7 (*CH*_*2(1)*_), 58.6 (*CH*_*2(3)*_), 57.9 (*CH*_*2(4)*_) ppm. Anal. calc. for C_48_H_72_Cu_3_F_24_N_17_O_4_P_4_·2H_2_O: C 28.81; H 2.72; N 16.80. Found: C 28.85; H 2.69; N 16.53. ESI-MS: *m*/*z* = 399 [C_16_H_16_CuN_8_O]^+^, 481 [C_16_H_16_Cu_2_FN_8_O]^+^, 1153 [C_32_H_32_Cu_3_F_12_N_16_O_2_P_2_]^+^.

#### H_3_LO–PF_6_ (**4**)

[Calix[4]imidazolium][hexafluorophosphate] (200.0 mg, 0.22 mmol) and Cu(ii) acetate monohydrate (84.0 mg, 0.47 mmol) were dissolved in 2 mL of dry MeCN in an inert atmosphere. The blue reaction mixture was stirred at r.t. for 5 min and then heated at 100 °C for 30 min. The colorless solution was cooled to r.t, forming a small amount of white precipitate (**2**). The suspension was transferred to a centrifuge vile and after centrifugation the clear colorless solution was decanted and precipitated with DCM (20 mL). The white precipitate was dissolved in MeCN (4 mL) and again precipitated with DCM (20 mL). The procedure was repeated twice and the resulting white powder was washed with DCM, and dried at 70 °C in air (130 mg, 0.170 mmol, 75% yield).^1^H NMR (400 MHz, DMSO-*d*_6_, 296 K): *δ* 9.81 (s, 1H, *CH*_*Im1a*_), 9.55 (s, 2H, *CH*_*Im2a*_), 7.97 (d, 2H, ^2^*J*_*HH*_ = 1.4 Hz, *CH*_*Im1b*_), 7.90 (s, 2H, *CH*_*Im2b*_), 7.77 (s, 2H, *CH*_*Im2c*_), 6.74 (s, 2H, *CH*_*Imox*_) 6.74 (s, 4H, *CH*_*2(1)*_), 6.04 (s, 4H, *CH*_*2(2)*_) ppm. ^1^H NMR (400 MHz, MeCN-*d*_3_, 296 K): *δ* 9.28 (s, 2H, *CH*_*Im2a*_), 9.25 (s, 1H, *CH*_*Im1a*_), 7.71 (d, 2H, ^2^*J*_*HH*_ = 1.4 Hz, *CH*_*Im1b*_), 7.63 (s, 2H, *CH*_*Im2b*_), 7.53 (s, 2H, *CH*_*Im2c*_), 6.59 (s, 2H, *CH*_*Imox*_), 6.53 (s, 4H, *CH*_*2(1)*_), 5.88 (s, 4H, *CH*_*2(2)*_) ppm. ^13^C{^1^H} NMR (101 MHz, MeCN-*d*_3_, 296 K): *δ* 153.8 (C=O), 139.0 (*C*_*Im1b*_), 133.8 (*C*_*Im1a*_), 125.0(*CH*_*Im1b*_), 124.1 (*CH*_*Im2b*_), 123.9 (*CH*_*Im2c*_), 112.6 (*CH*_*Imox*_), 59.90 (*CH*_*2(1)*_), 57.0 (*CH*_*2(2)*_) ppm. Anal. calc. for C_16_H_19_F_18_N_8_OP_3_: C 24.82; H 2.47; N 14.47. Found: C 24.39; H 2.19; N 14.08. ESI-MS: *m*/*z* = 483 [C_16_H_18_F_6_N_8_OP]^+^, 629 [C_16_H_19_F_12_N_8_OP_2_]^+^.

#### CuH_2_LO–PF_6_ (**5**)

[Calix[4]imidazolium]-[hexafluorophosphate] (10.0 mg, 0.011 mmol) and Cu(ii) acetate monohydrate (4.40 mg, 0.22 mmol) were dissolved in 0.4 mL of dry and degassed MeCN-*d*_3_ in an inert atmosphere. The blue reaction mixture was stirred at r.t. for 5 min and then heated at 80 °C for 5 min. The colorless solution was cooled to r.t. The clear solution contained an approx. 1 : 1 mixture of 4 and 5. ^1^H NMR (400 MHz, MeCN-*d*_3_, 296 K): *δ* 9.14 (s, 2H, *CH*_*Im2a*_), 7.58 (s, 2H, *CH*_*Ima*_), 7.45 (s, 2H, *CH*_*Imb*_), 7.58 (s, 2H, *CH*_*ImCu*_), 6.54 (s, 2H, *CH*_*Imox*_), 6.52 (d, 2H, ^2^*J*_*HH*_ = 14.3 Hz, *CH*_*2(a)*_), 6.11 (d, 2H, ^2^*J*_*HH*_ = 14.3 Hz, *CH*_*2(a)*_), 6.06 (d, 2H, ^2^*J*_*HH*_ = 14.3 Hz, *CH*_*2(b)*_), 5.60 (d, 2H, ^2^*J*_*HH*_ = 14.3 Hz, *CH*_*2(b)*_) ppm. ^13^C{^1^H} NMR (101 MHz, MeCN-*d*_3_, 296 K): *δ* 187.9 (*C*_*carbene*_), 154.2 (C=O), 138.3 (*C*_*ImH*_), 123.2 (*CH*_*ImCu*_), 123.2 (*CH*_*Ima/b*_), 123.2 (*CH*_*Ima/b*_), 112.5 (*CH*_*Imox*_), 62.5 (*CH*_*2(a/b)*_), 56.5 (*CH*_*2(a/b)*_) ppm.

## Conflicts of interest

There are no conflicts to declare.

## Supplementary Material

Supplementary information

Crystal structure data
