# Supplementary material for: A bench stable formal Cu(iii) N-heterocyclic carbene accessible from simple copper(ii) acetate
Source: Chem Sci. 2018 Sep 14;9(43):8307–14. doi: 10.1039/c8sc01834k (PMC6240905; doi:10.1039/c8sc01834k)
Supplement: Supplementary file 1 [file SC-009-C8SC01834K-s001.pdf]

Electronic Supplementary Material (ESI) for Chemical Science.
This journal is © The Royal Society of Chemistry 2018

Electronic Supplementary Information

A bench stable formal Cu(III) N-heterocyclic carbene accessible from simple copper(II) acetate

Zohreh S. Ghavami,[b,‡] Markus R. Anneser,[a,‡] Felix Kaiser,[a] Philipp J. Altmann,[a] Benjamin J. Hofmann,[a] Jonas F. Schlagintweit,[a] Gholamhossein Grivani,[b] and Fritz E. Kühn*,[a]

[a]Molecular Catalysis, Catalysis Research Center and Faculty of Chemistry, Lichtenbergstrasse 4, Technische Universität München, D-85747 Garching bei München, Germany.

[b]School of Chemistry, Damghan University, Damghan 36715-364, Iran

Table of contents:

1.	Single Crystal X-ray Diffraction	2
2.	Experimental	6
3.	ESI-MS-Data	8
4.	^{1}H NMR and ^{13}C NMR Spectra of Compounds 1 to 5	9
5.	^{1}H NMR Kinetics	20
6.	Literature	26

1. Single Crystal X-ray Diffraction

General crystallographic details

Data were collected on an X-ray single crystal diffractometer equipped with a CCD detector (Bruker D8 Venture Duo IMS, APEX III, κ-CCD, λ = 0.71073 Å) equipped with a Helios optic monochromator (**1**, **3**) or a rotating anode (Bruker TXS) with $MoK_α$ radiation (λ = 0.71073 Å) and a Helios optic monochromator (**2**, **4**) by using the APEX software package.[1] The measurements were performed on a single crystal coated with perfluorinated ether. The crystal was fixed on top of a microsampler and transferred to the diffractometer. The crystal was frozen under a stream of cold nitrogen. A matrix scan was used to determine the initial lattice parameters. Reflections were merged and corrected for Lorentz and polarization effects, scan speed, and background using SAINT.[2] Absorption corrections, including odd and even ordered spherical harmonics were performed using SADABS.[3] Space group assignments were based upon systematic absences, E statistics, and successful refinement of the structures. Structures were solved by direct methods with the aid of successive difference Fourier maps, and were refined against all data using SHELXLE[3] in conjunction with SHELXL-2014[4]. Hydrogen atoms were assigned to ideal positions and refined using a riding model with an isotropic thermal parameter 1.2 times that of the attached carbon atom (1.5 times for methyl hydrogen atoms). If not mentioned otherwise, non-hydrogen atoms were refined with anisotropic displacement parameters. Full-matrix least-squares refinements were carried out by minimizing $\Sigma w(Fo^2-Fc^2)^2$ with SHELXL-97[5] weighting scheme. Neutral atom scattering factors for all atoms and anomalous dispersion corrections for the non-hydrogen atoms were taken from International Tables for Crystallography.[6] Images of the crystal structures were generated by PLATON.[7]

Special crystallographic details

Compound **1**:

The hexafluorophosphate counterions as well as the weakly coordinated diethyl ether are heavily disordered and partly located at special positions, leading to bigger ellipsoids in these cases and furthermore increased R-values.

Compound **3**:

The copper atoms within the sandwich complex and the whole sandwich complex itself are rotationally disordered. Due to the high electron count of copper compared to the organic ligand frame the ligand related disorder cannot be resolved. Only two split layers with occupancy ratios of approximately 82:18 and 94:6 for copper atoms Cu1 and Cu3 have been included into the refinement. All remaining electron densities in the central complex plane were smaller and have thus been left "as is". One disordered, co-crystallized molecule of dichloromethane is located centrally in the bowl-shaped cavity, generated by the ligand at the ends of the complex. Due to disorder the atoms were refined isotropically. These circumstances, combined with the counter ion and related disorder also lead to the comparatively high R-values.

Preparation and characterisation of compound 2.

Although **2** is easily separated from a mixture with **1**, due to its insolubility in MeCN, yields are very low (~10%) using synthetic protocols for **1** under anaerobic conditions. Thus, **2** being presumably formed during the reaction by Cu(I) acetate, (generated *in situ*; *vide infra*), a direct synthesis for **2** with Cu(I) acetate was developed, compensating the lack of internal base by the addition of external NBu_4OAc. Eventually, **2** is best prepared by mixing H_4LX_4, Cu(I) acetate and NBu_4OAc in a stoichiometric fashion (1:2:2) and heating for 30 min at 100°C in DMSO under an inert gas atmosphere (equation II).

$$\text{(S-I)}\quad 2\ H_4LX_4 + 4\ \text{Cu(I)OAc} + 4\ NBu_4OAc \longrightarrow \underset{\mathbf{2}}{\text{Cu(I)}_4L_2X_4} + 4\ NBu_4X + 8\ HOAc$$

It has to be noted, that presence of oxygen during the synthesis leads to significantly reduced yields of **2**, since the formation of initially **1** and later the oxidized species **3** is favored under these conditions. After removal of the solvent, the crude product can be easily purified by washing with acetonitrile, yielding **2** as a white powder in good yields (~75 %). **2** is air stable in solid state, although it slowly reacts to **1** and **3**, when dissolved in DMSO and exposed to air (see 5.3).

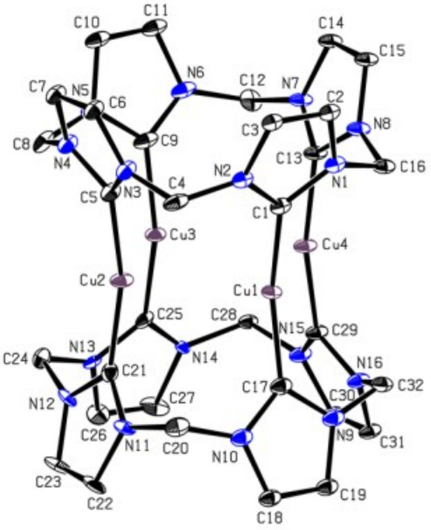

Figure S1. X-ray structure of the cationic fragment of compound **2**. Hydrogen atoms and four PF_6^- anions are omitted for clarity. Thermal ellipsoids are shown at a 50% probability level. Selected bond lengths (Å) and angles (deg): Cu1−C1: 1.911(11), Cu1−C17: 1.9018(12); Cu2−C5: 1.912(13), Cu2−C21: 1.904(13), Cu3−C9: 1.906(11), Cu3−C25: 1.918(11); Cu4-C13: 1.907(12), Cu4−C29: 1.900(12), Cu1−Cu4: 3.052(2), Cu1−Cu2: 3.070(2), Cu2−Cu3: 3.040(2), Cu3−Cu4: 3.062(2); C1-Cu1-Cu17: 160.9(5), C5-Cu2-C21: 163.4(5), C9-Cu3-C25: 159.0(5), C13-Cu4-C29: 163.2(6). Cu1-Cu2-Cu3: 90.1(3), Cu2-Cu3-Cu4: 90.2(3), Cu3-Cu4-Cu1: 90.0(2), Cu4-Cu1-Cu2: 89.8(3).

The single crystal structure of **2** is very similar to those of its higher homologues Ag and Au (Fig. S1). The Cu-C bond lengths vary between 1.90 and 1.92 Å (Ag/Au: 2.09/2.03 Å) and the C-Cu-C angle lies between 159 and 163° (Ag/Au: 166/165°). The Cu-Cu distances range between 3.04 and 3.07 Å (Ag/Au: 3.20/3.15 Å), which is considerably longer than the sum of the respective van der Waals radii of 2.80 Å (Ag/Au: 3.44/3.32 Å).[8] Thus, the slight inward displacement from an ideal linear coordination of the Cu(I) ions, might be attributed to strain from the ligand moieties and not to any significant Cu(I)···Cu(I) interactions. The ^{1}H-NMR signals at 7.71 ppm and 6.99/6.40 ppm and the ^{13}C-NMR signals at 178.8, 122.1 and 64.0 ppm coincide very well with those of literature known Cu(I) *bis*(NHC) complexes.[9,10]

Preparation and characterisation of compound 3.

The Cu_3LO_2 compound **3** can also be obtained by a variety of different methods, though it is most favorably formed by mixing H_4LX_4, Cu(II) acetate and NBu_4OAc in a stoichiometric fashion (1:2:2; equation III). In this way, **3** can be isolated in very good yields of >90% and in very high purity.

$$\text{(S-II)} \quad 2\ H_4LX_4 + 4\ Cu(II)(OAc)_2 + 4\ NBu_4OAc \longrightarrow \underset{\mathbf{3}}{Cu(I)_3LO_2X_3} + Cu(I)X + 4\ NBu_4X + 8\ HOAc + 2\ Ac_2O$$

It is noteworthy that NBu_4OAc is crucial for a clean formation of **2** and **3**. Other acetate bases, such as NaOAc diminish the yield and purity of the respective compounds significantly, probably due to their worse solubility in DMSO/MeCN. Unlike **1** or **2**, **3** is a slightly yellow powder. The crystal structure (Fig. S2) reveals a highly unusual structure for compound **3.** The outer (Cu1/3) and inner Cu(I)-ions (Cu2) show two distinct coordination spheres: The first are coordinated in a highly distorted trigonal planar fashion by two C- and one O-atom, while the latter depicts a distorted tetrahedral coordination by two C- and two O-atoms. While the Cu-C distances are well within the established range for Cu(I)NHC (1.91-1.93 Å), the Cu-O distances are comparatively large (2.29-2.70 Å).

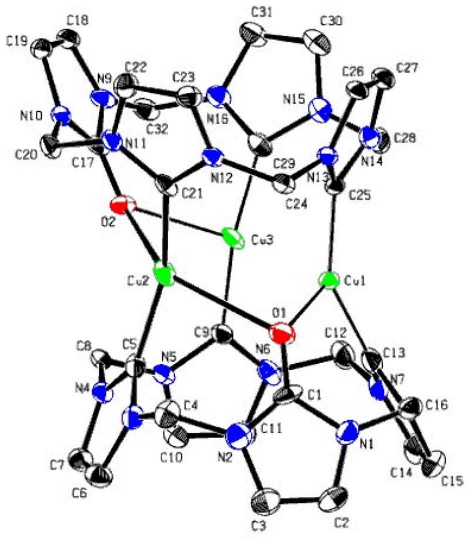

Figure S2. X-ray structure of the cationic fragment of compound **3**. Hydrogen atoms and three OTf^{-} anions are omitted for

clarity. Thermal ellipsoids are shown at a 30% probability level. Selected bond lengths (Å) and angles (deg): Cu1−Cu2: 3.3207(3), Cu1−Cu3: 2.8764(3); Cu2−Cu3: 3.3398(3), Cu2−O1: 2.7003(2), Cu2−O2: 2.6238(2), Cu3−O2: 2.396(3); Cu1-O1: 2.2924(2), Cu2−C21: 1.9288(2), Cu2−C5: 1.9260(2), Cu1−C25: 1.9298(2), Cu1−C13: 1.9289(2), Cu3−C9: 1.9157(2); Cu3−C29: 1.9217(2); C1-O1: 1.2138(1), C17-O2: 1.2375(1), C13-Cu1-C25: 150.1(8), C5-Cu2-C21: 157.1(7), C9-Cu3-Cu29: 153.0(7), Cu1-Cu2-Cu3: 51.0(3), Cu2-Cu1-Cu3: 64.9(3), Cu1-Cu3-Cu2: 64.2(3), Cu2-O2-Cu1: 83.3(4), Cu2-O2-Cu3: 83.0(4), O1-Cu2-O2: 137.7(4), C5-Cu2-C21: 157.1(7), O2-Cu3-C29: 93.4(6), O2-Cu2-C9: 104.2(6), O1-Cu1-C25: 101.8(6), O1-Cu1-C13: 97.0(7).

Despite being asymmetric in the solid state, the ^{1}H-NMR of **3** indicates fluxional behavior in solution, thus only half of the expected signals are observed (see 5.4). The three $C_{carbene}$ signals are located very close to 180 ppm (180.5 to 179.9 ppm), similar to **2** and other Cu(I)NHCs. The very characteristic signals of the oxidized NHC can be found at 151.8, 111.9 and 111.8 ppm, in good agreement with related structures.[11,12,13]

Complex **3**, especially when compared to **2**, displays very high stability towards air even in solution. It is completely stable towards oxygen and moisture up to 100°C in DMSO solution and in the solid state. Also, prolonged storage at 70°C results in no visual or spectroscopic changes. However, **3** is mildly sensitive to strong Brønsted acids such as hydrochloric- and triflic acid and treatment with those acids results in the smooth formation of the oxidized imidazolium salt **4** and the respective Cu(I)X salts. This process is easily reversible by addition of Cu(I) acetate and additional base.

2. Experimental

2.1. H_3LO-Cl chloride (4-Cl): [Calix[4]imidazolium][hexafluorophosphate] (500 mg, 0.55 mmol) and Cu(II)OAc (230 mg, 1.16 mmol) are dissolved in 5 mL of MeCN under an argon atmosphere. The reaction mixture is stirred at r.t. for 5 min. and then heated to 100 °C for 30 min. During this time the color changes from blue over green to yellow. After cooling to r.t. a white precipitate forms and the clear yellow solution is separated by centrifugation on air. DCM is added to the solution to precipitate a yellow powder, which is separated by centrifugation. The powder is dissolved MeCN and 5 mL of concentrated hydrochloric acid were added, leading to the formation of an oily yellow precipitate. It is separated, washed with MeCN and dissolved in the minimum amount of DMSO. The clear yellow solution is precipitated with MeCN. The yellow solution is separated from the white precipitate. This is repeated once. Successively the white precipitate is washed twice with DCM and dried at 70°C on air to obtain a white powder (110 mg, 45 % yield).

^{1}H NMR (400 MHz, DMSO-d_6, 296 K): δ 9.90 (s, 1H, CH_{Im1a}), 9.60 (s, 2H, CH_{Im2a}), 8.10 (d, 2H, $^2J_{HH}$ = 1.5 Hz, CH_{Im1b}), 8.03 (s, 2H, CH_{Im2b}), 7.85 (s, 2H, CH_{Im2c}), 6.83 (s, 2H, CH_{Imox}) 6.80 (s, 4H, $CH_{2(1)}$), 6.06 (s, 4H, $CH_{2(2)}$) ppm. ^{13}C{^{1}H} NMR (101 MHz, DMSO-d_6, 296 K): δ 150. 28 (C=O), 138.01 (C_{Im1b}), 133.21 (C_{Im1a}), 123.70 (CH_{Im1b}), 122.74 (CH_{Im2b}), 122.49 (CH_{Im2c}), 111.62 (CH_{Imox}), 58.44 ($CH_{2(1)}$), 55.23 ($CH_{2(2)}$) ppm. Anal. Calcd for $C_{16}H_{19}Cl_3N_8O$: C 43.11; H 4.30; N 25.14. Found: C 42.78; H 4.11; N 24.53.

2.2. Preparation of ^{18}O-Labeled $Cu(OAc)_2$: $H_2{}^{18}O$ (98%,100 µL, 4.50 mmol) is mixed with acetic anhydride (60.0 µL, 0.54 mmol) and stirred for four days under inert atmosphere. Cu(II)O (30.0 mg, 0.38 mmol) is added to the ^{18}O-enriched acetic acid and the black suspension is heated for 24 h at 80°C. Thereafter the blue solution is dried for 4 h at 80°C in high vacuum resulting in a bluish green solid ($Cu(OAc)_2$: 59.0 mg, 0.33 mmol).

2.3. Preparation of ^{18}O-Labeled Cu_3Lox_2 hexafluorophosphate (3-^{18}O):

2.3.1. ^{18}O Labeling of 3 with Cu(II)(^{18}OAc)$_2$: [Calix[4]imidazolium][hexafluorophosphate] (50.0 mg, 0.055 mmol), Cu(II)(^{18}OAc)$_2$ (16.0 mg, 0.09 mmol) and NBu_4OAc (36.0 mg, 0.12 mmol) are dissolved in 1 mL of dry DMSO under inert atmosphere. The blue reaction mixture is stirred at r.t. for 5 min and then heated at 100 °C for 10 min. The clear green solution is evaporated (100°C, Schlenk vacuum), yielding a green solid. The residue is dissolved with MeCN (2 mL) and precipitated with DCM (8 mL). After centrifugation a yellow solid separates from the slight green supernatant solution. The supernatant solution is decanted off and the procedure is repeated thrice with the remaining solid. Successively the precipitate is washed twice with DCM and dried at 70°C on air to obtain a light yellow powder (33 mg, 0.51 mmol, 91 % yield). ESI MS analysis confirms an ^{18}O-content of 28 ± 1.8 %.

2.3.2. ^{18}O Labeling of 3 with $H_2{}^{18}O$: [Calix[4]imidazolium][hexafluorophosphate] (50.0 mg, 0.055 mmol), $Cu(II)(OAc)_2$ (16.0 mg, 0.09 mmol) and NBu_4OAc (36.0 mg, 0.12 mmol) are dissolved in 1 mL of dry DMSO mixed with 10% $H_2{}^{18}O$ (114.0 µL, 6.37 mmol) under inert atmosphere. Immediately the blue reaction mixture is stirred at r.t. for 5 min. and then heated at 100 °C for 10 min. The clear slight green solution is evaporated (100°C, Schlenk vacuum), yielding a green solid. The residue is dissolved with MeCN (2 mL) and precipitated with DCM (8 mL). After centrifugation a yellow solid separates from the slight green supernatant solution. The supernatant solution is decanted off and the procedure is repeated thrice with the remaining solid. Successively the precipitate is washed twice with DCM and dried at 70°C on air to obtain a light yellow powder (25 mg, 0.039 mmol, 71 % yield). ESI MS analysis confirms an ^{18}O-content of 0.4 ± 1.4 %.

2.3.3. ^{18}O Labeling of 3 with $^{18}O_2$: [Calix[4]imidazolium][hexafluorophosphate] (100.0 mg, 0.11 mmol), $Cu(II)(OAc)_2$ (44.0 mg, 0.24 mmol) and NBu_4OAc (72.0 mg, 0.23 mmol) are dissolved in 2 mL of dry DMSO and immediately frozen in liquid nitrogen. The argon atmosphere is removed and replaced by 1 bar of $^{18}O_2$ (98%, approx. 20 mL, 0.82 mmol). The closed tube is stirred in an r.t. water bath for 5 min. and then heated at 100 °C for 10 min. The intense green, clear solution is evaporated (100°C, Schlenk vacuum), yielding a dark green solid. The residue is dissolved with MeCN (2 mL) and precipitated with DCM (8 mL). After centrifugation a yellow solid separates from the intense green supernatant solution. The supernatant solution is decanted off and the procedure is repeated thrice with the remaining solid. Successively the precipitate is washed twice with DCM and dried at 70°C on air to obtain a light yellow powder (65 mg, 0.050 mmol, 91 % yield). ESI MS analysis confirms an ^{18}O-content of 1.6 ± 1.2 %.

2.4. Preparation of ^{18}O-Labeled H_3LO hexafluorophosphate (4-^{18}O): [Calix[4]imidazolium][hexafluorophosphate] (50.0 mg, 0.055 mmol), $Cu(II)(^{18}OAc)_2$ (21.0 mg, 0.12 mmol) are dissolved in 1 mL of dry MeCN under inert atmosphere. The blue reaction mixture is stirred at r.t. for 5 min and then heated at 100 °C for 30 min. The colorless solution is cooled to r.t upon which a small amount of white precipitate forms. The suspension is transferred to a centrifuge tube and after centrifugation the clear colorless solution is decanted and precipitated with DCM (8 mL). The white precipitate is dissolved in MeCN (2 mL) and again precipitated with DCM (8 mL). This is repeated twice and the resulting white powder is washed with DCM, and dried at 70°C on air in a drying oven (32 mg, 0.042 mmol, 75 % yield). ESI MS analysis confirms an ^{18}O-content of 53 ± 0.7 %.

3. ESI-MS Data

All Electrospray ionization (ESI) mass spectrometry (MS) data were acquired on a *Thermo FisherUltimate 3000*. The sample had an approximate concentration of 0.1 mg/ml of the substrate dissolved in HPLC-grade Acetonitrile.

Table S1: Collected raw data used for the calculation of the ^{18}O-Enrichment in ^{18}O-**3** and ^{18}O-**4**. (The Reference column provides the mean results of three runs for the unlabeled compounds. Runs 1-3 represent the original values of three runs measured form the same sample of ^{18}O-labeled substrate.)

m/z	Unlabeled	^{18}O-labeled	^{18}O-labeled	^{18}O-labeled	Mean ^{18}O-enrichment	Standard deviation
**2.3.1**	**Reference**	**Run 1**	**Run 2**	**Run 3**	27.9%	1.76%
399,24	100,00%	100,00%	100,00%	100,00%		
401,20	44,47%	82,49%	88,29%	73,45%		
403,32	0,46%	18,75%	19,84%	20,76%		
**2.3.2**						
399,24	100,00%	100,00%	100,00%	100,00%	0.39%	1.36%
401,20	44,47%	40,58%	46,44%	46,61%		
403,32	0,46%	1,73%	0,71%	0,55%		
**2.3.3**						
399,24	100,00%	100,00%	100,00%	100,00%	1.60%	1.16%
401,20	44,47%	48,36%	44,07%	47,86%		
403,32	0,46%	0,53%	0,47%	0,65%		
**2.4**						
483,15	100,00%	100,00%	100,00%	100,00%	53.4%	0.74%
485,16	2,75%	114,30%	121,91%	123,83%		
487,19	1,79%	4,53%	3,56%	4,86%		

4. ¹H NMR and ¹³C NMR Spectra of Compounds 1 to 5

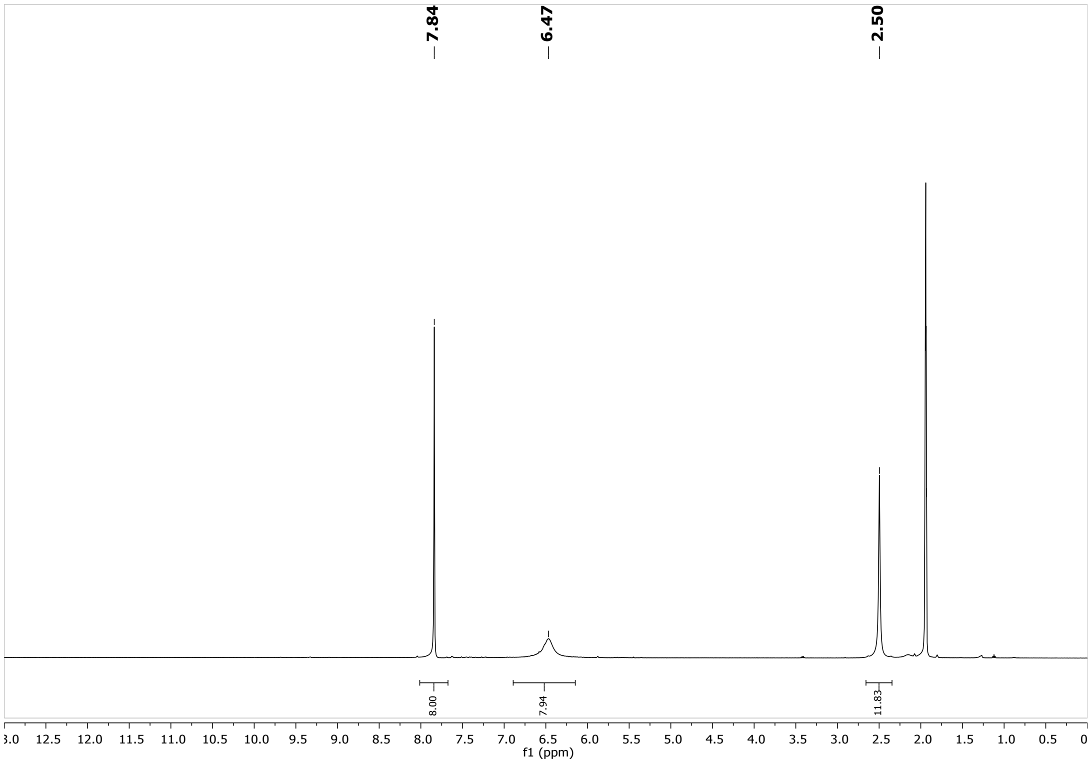

Fig. S3: Processed ¹H-NMR of **1** in MeCN-d_3.

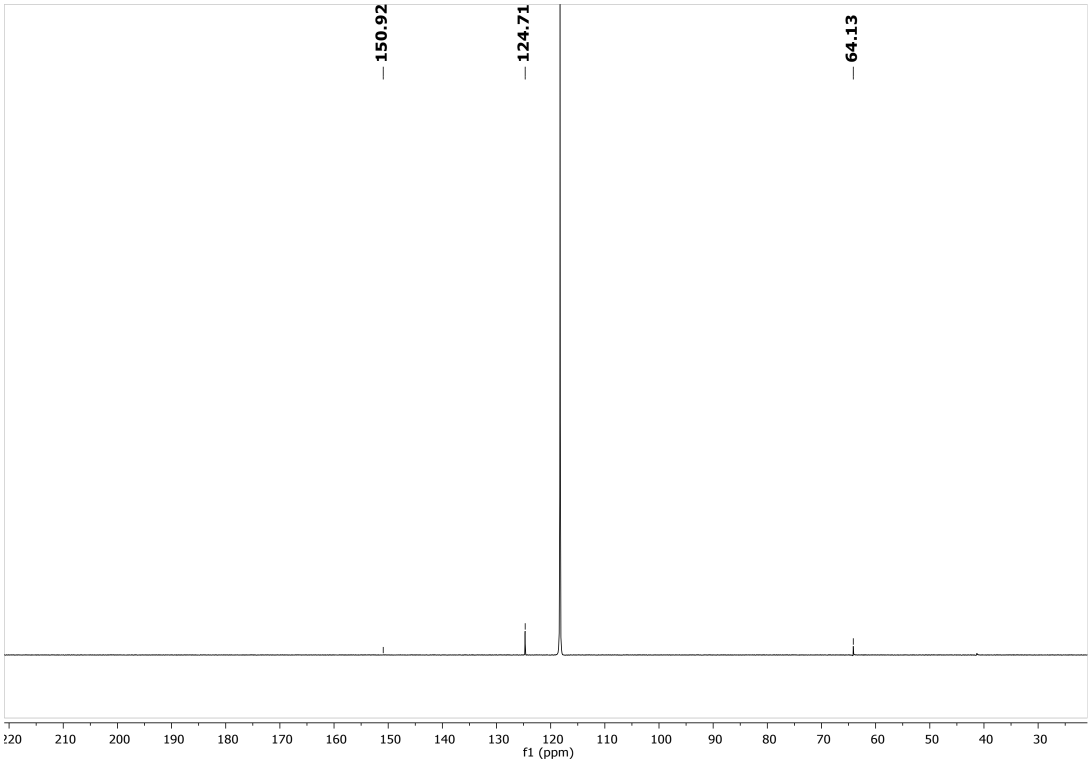

Fig. S4: Processed ¹³C-NMR of **1** in MeCN-d_3.

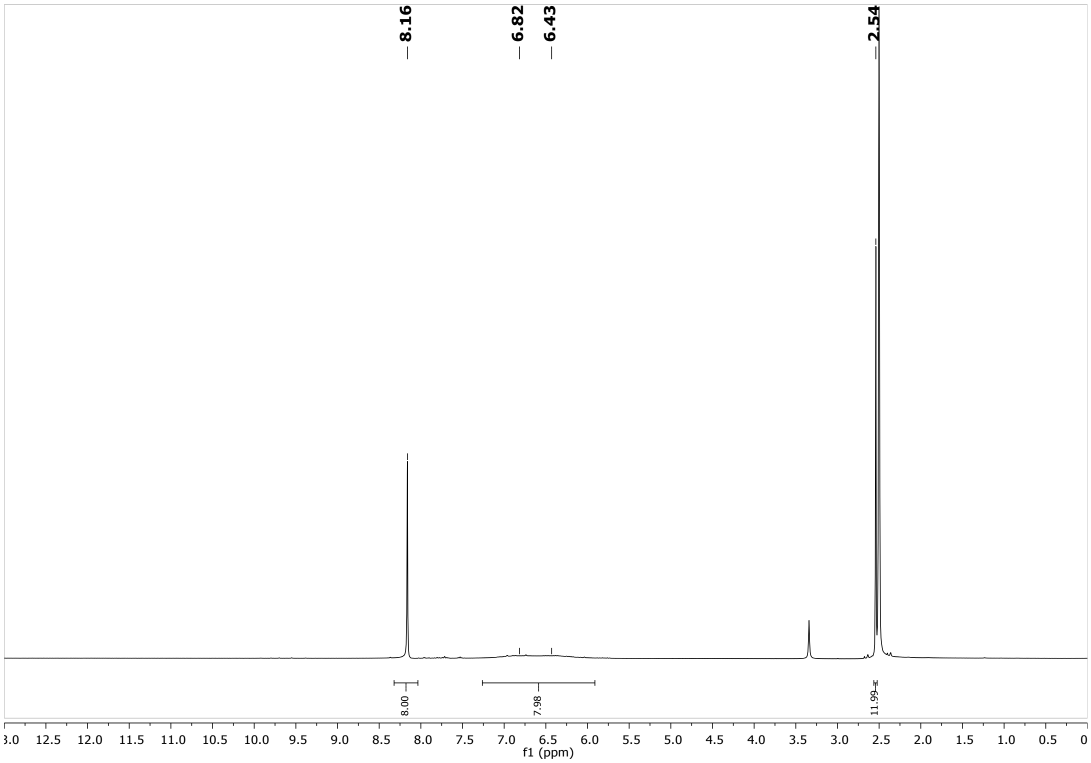

Fig. S5: Processed ^{1}H-NMR of **1** in DMSO-d_6.

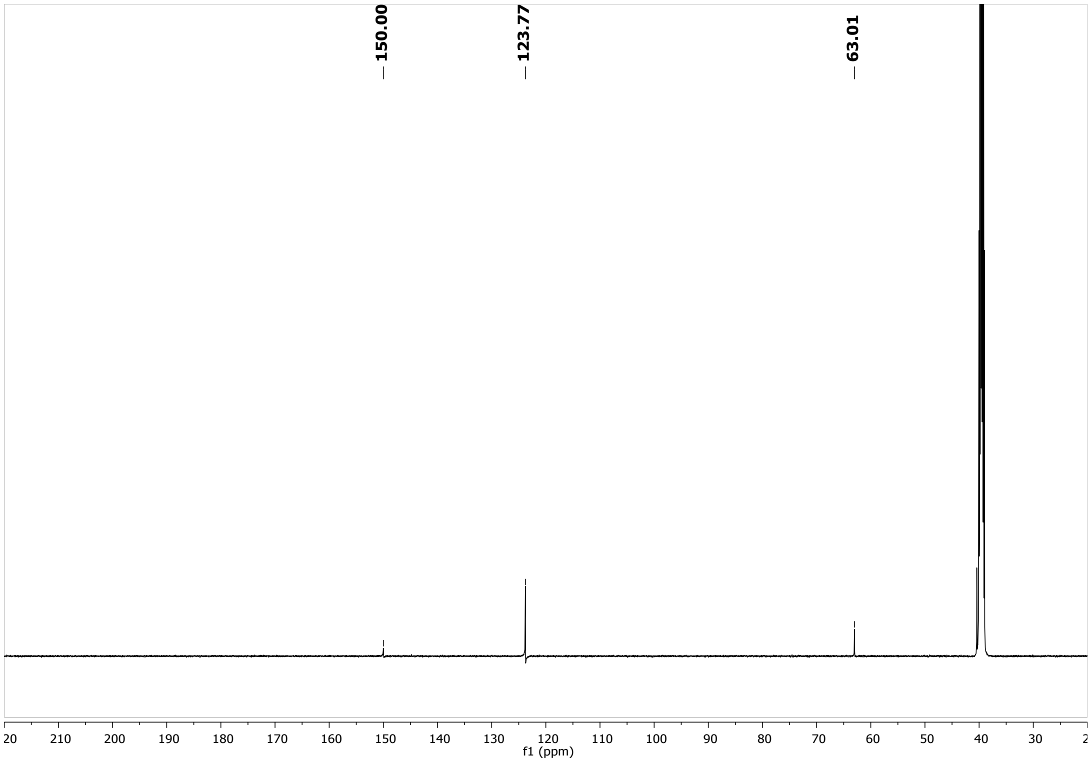

Fig. S6: Processed ^{13}C-NMR of **1** in DMSO-d_6.

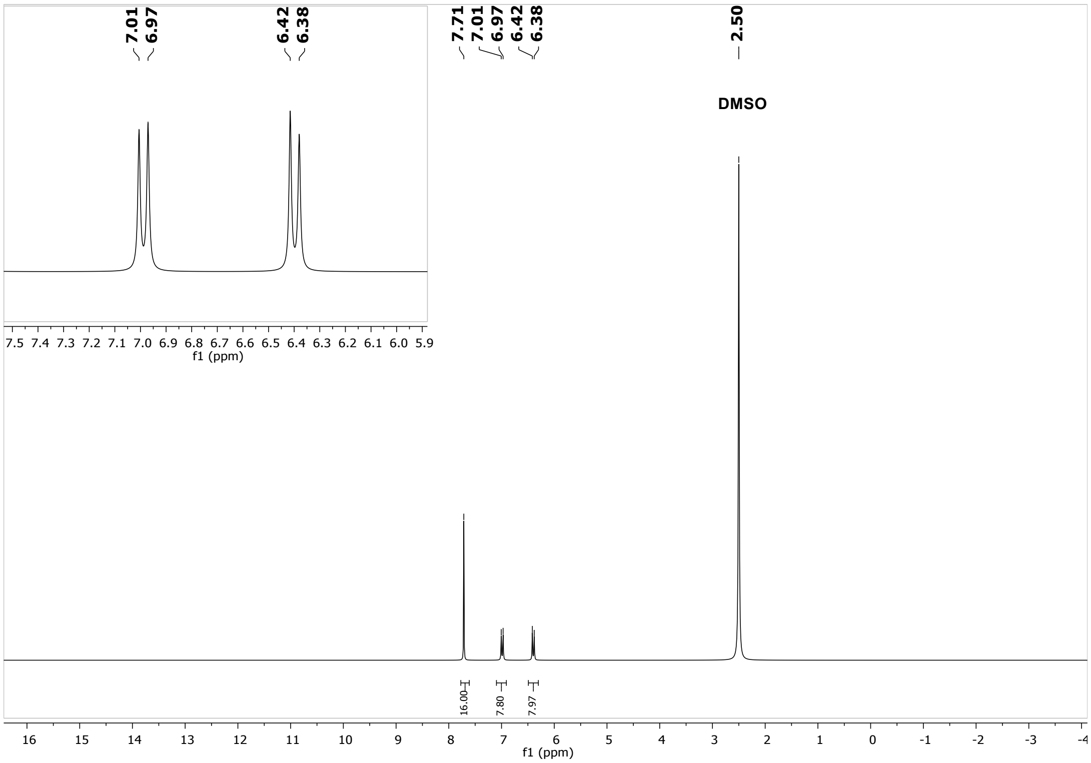

Fig. S7: Processed ^{1}H-NMR of **2** in DMSO-d_6.

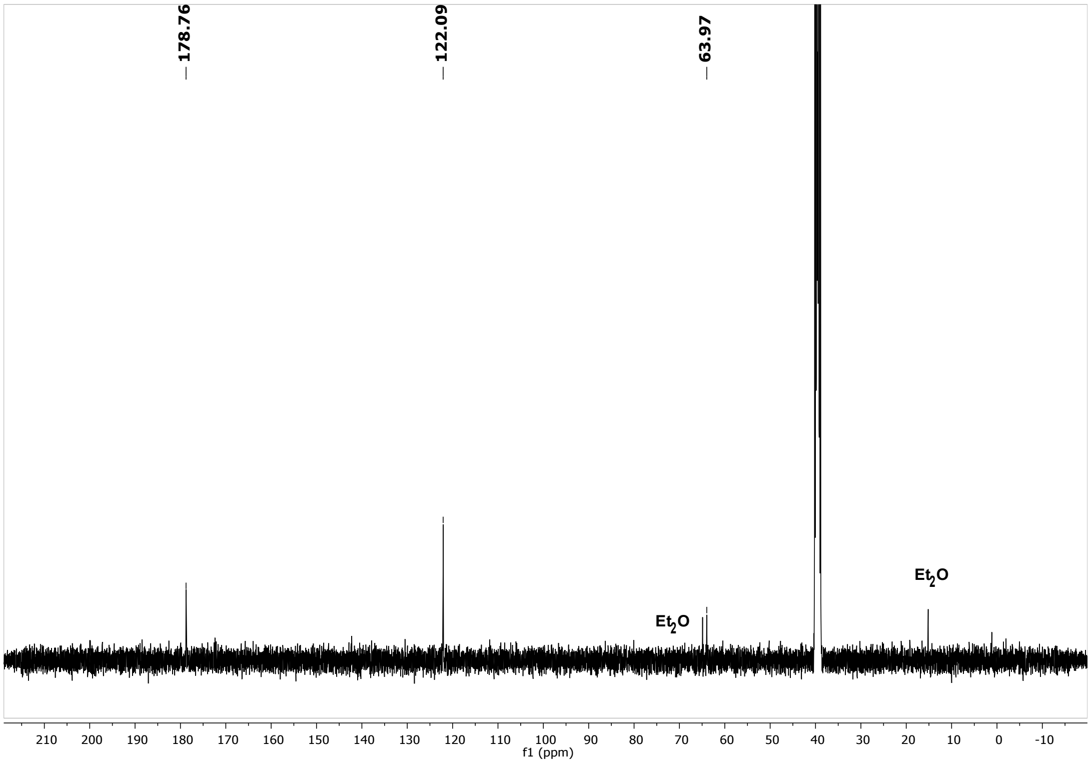

Fig. S8: Processed ^{13}C-NMR of **2** in DMSO-d_6.

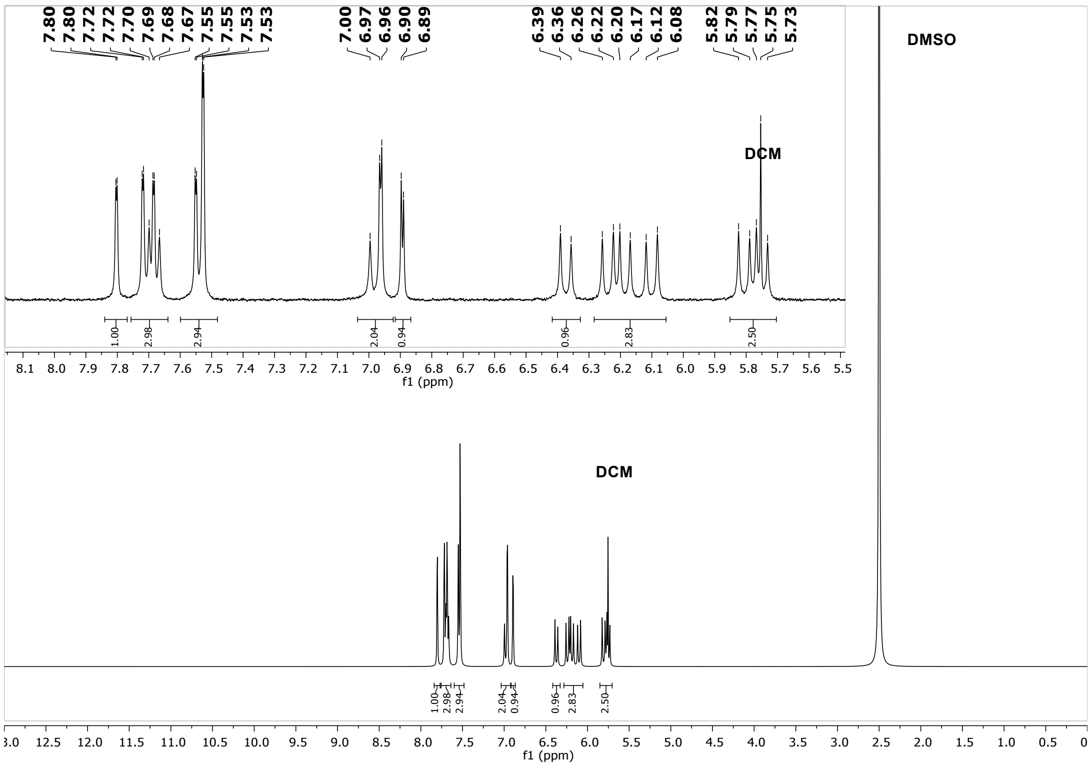

Fig. S9: Processed ¹H-NMR of **3-PF$_6$** in DMSO-d$_6$.

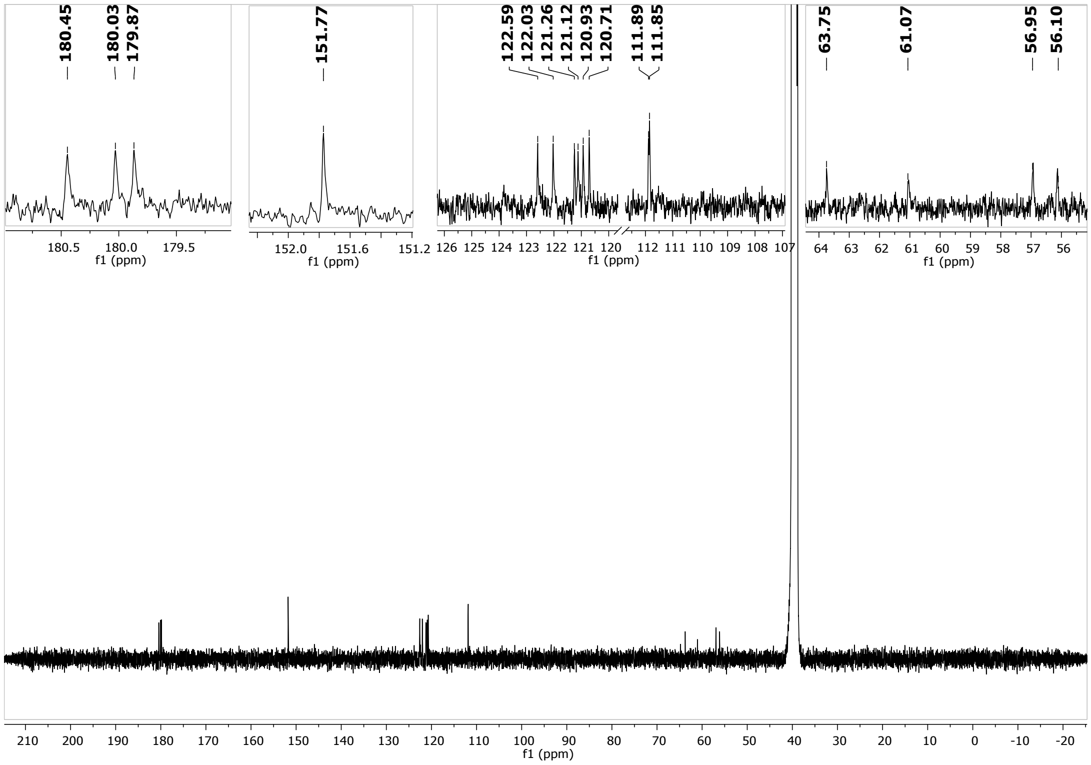

Fig. S10: ¹³C-NMR of **3-PF$_6$** in DMSO-*d$_6$*.

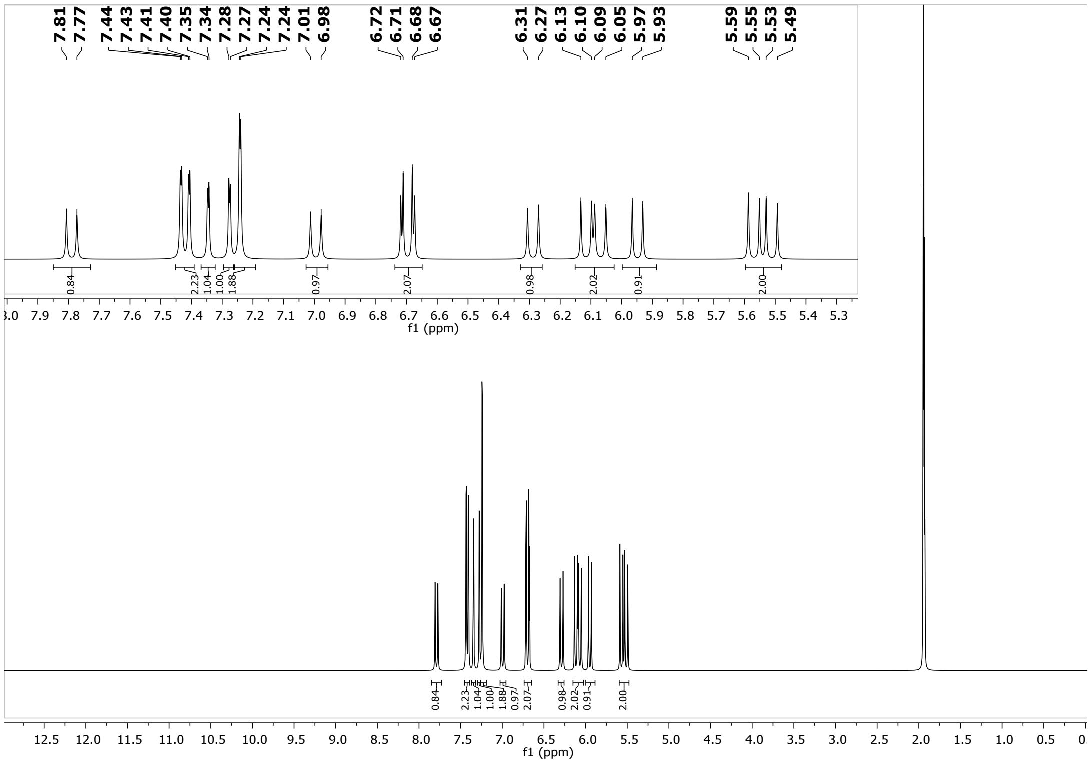

Fig. S11: Processed ^{1}H-NMR of **3-PF₆** in MeCN-d_3.

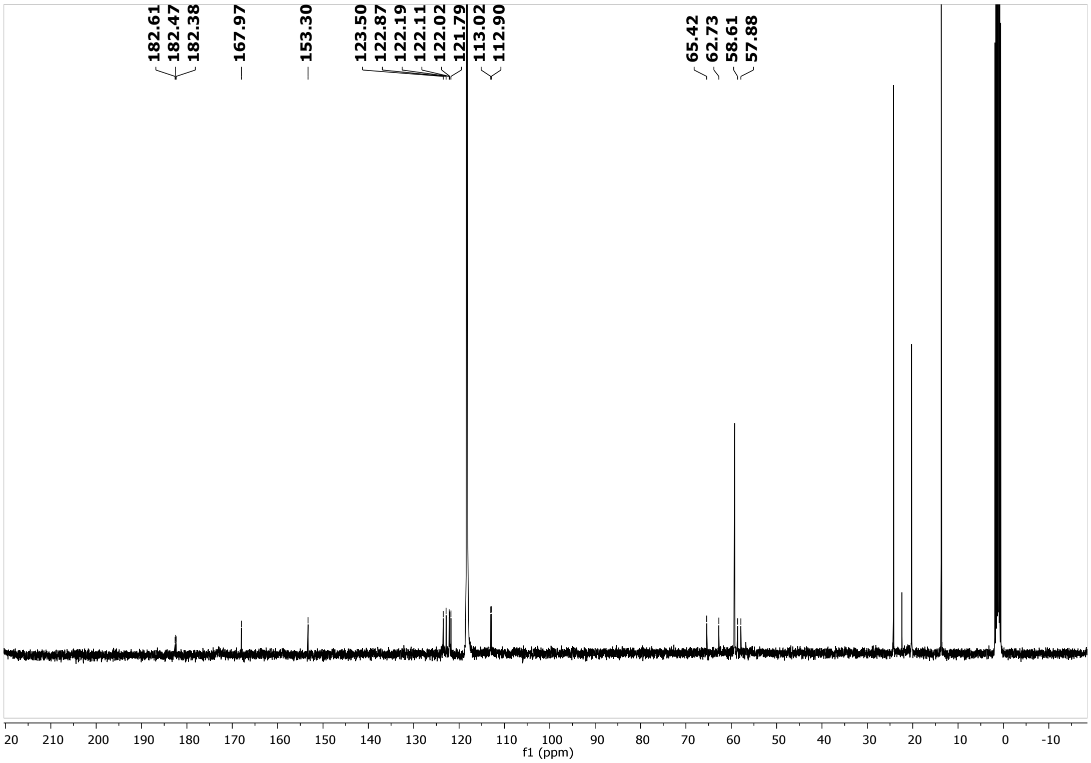

Fig. S12: Processed ^{13}C-NMR of **3-PF₆** in MeCN-d_3.

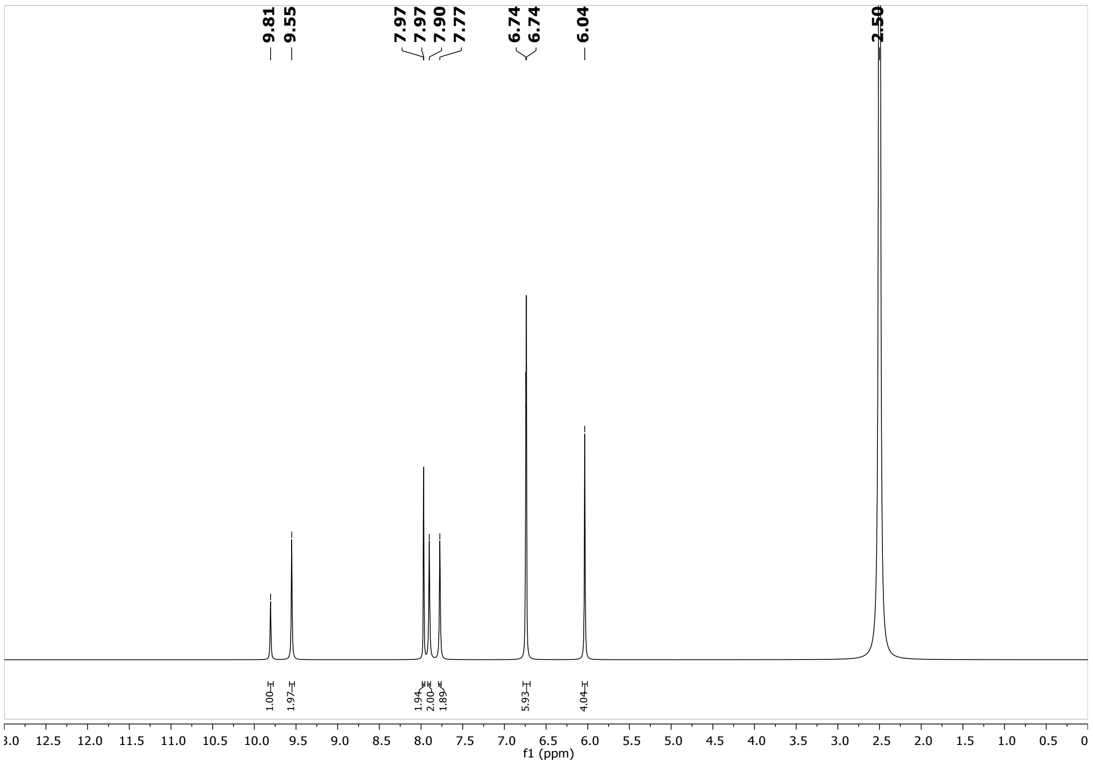

Fig. S13: Processed ^{1}H-NMR of **4-PF$_6$** in DMSO-d_6.

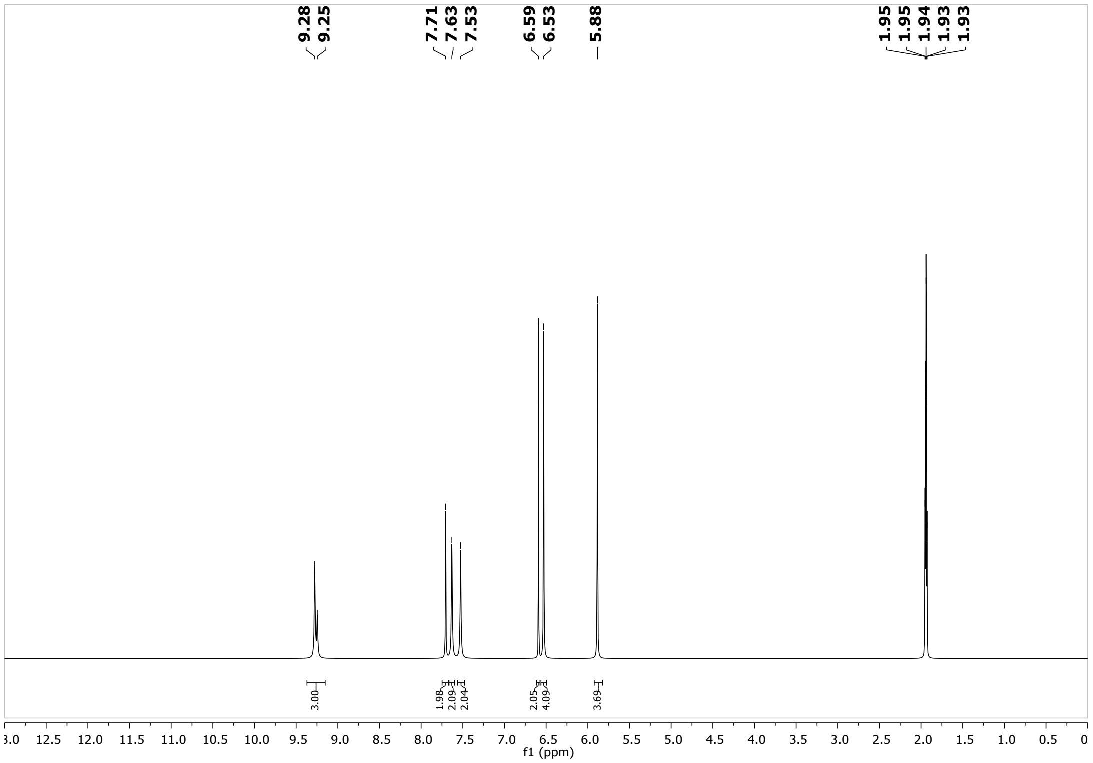

Fig. S14: Processed ^{1}H-NMR of **4-PF$_6$** in MeCN-d_3.

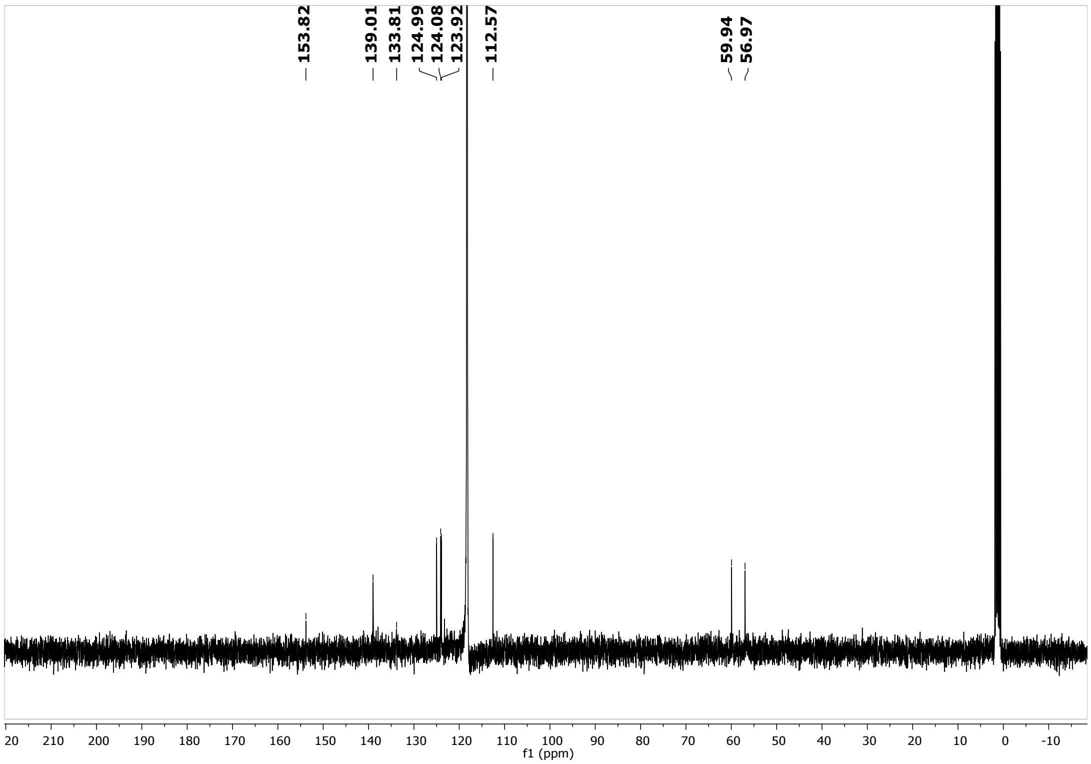

Fig. S15: Processed ^{13}C-NMR of **4-PF$_6$** in MeCN-d_3.

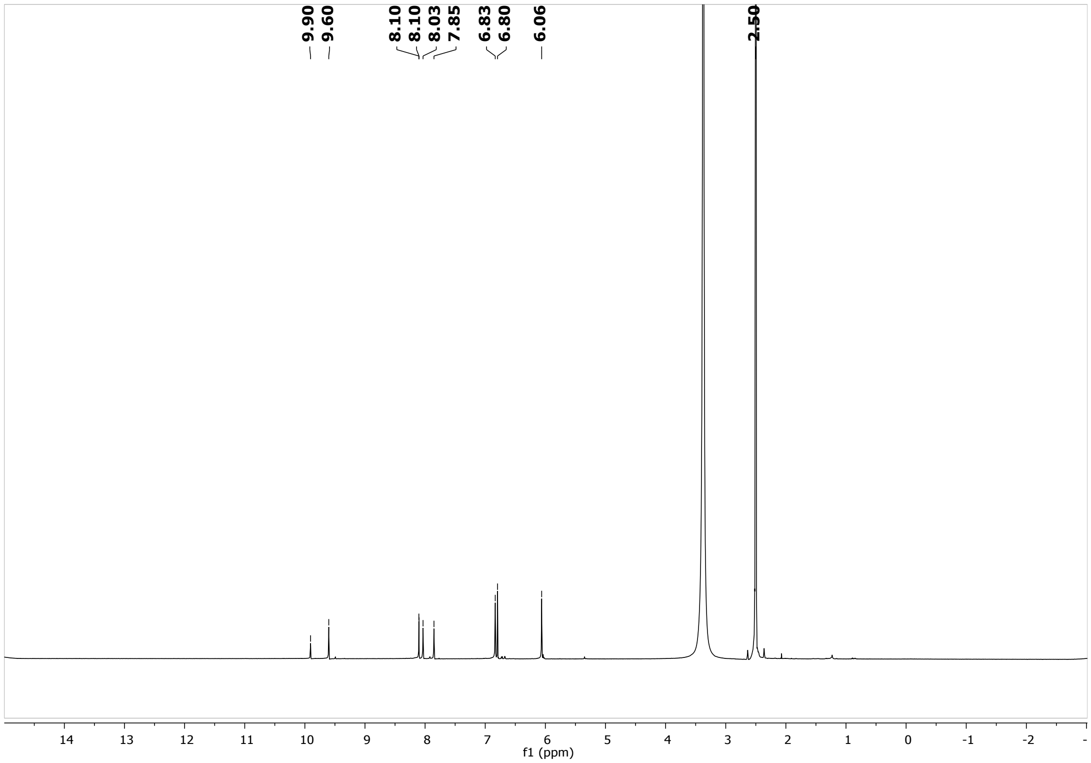

Fig. S16: Processed ^{1}H-NMR of **4-Cl** in DMSO-d_6.

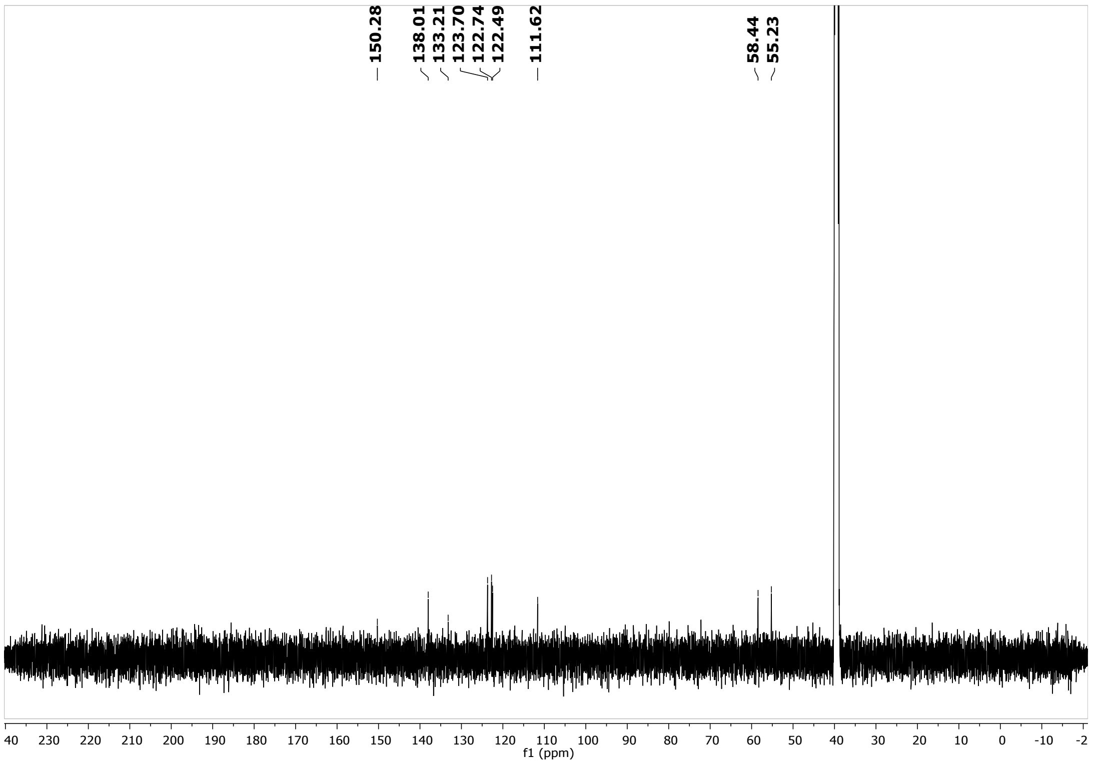

Fig. S17: Processed ^{13}C-NMR of **4-Cl** in DMSO-d_6.

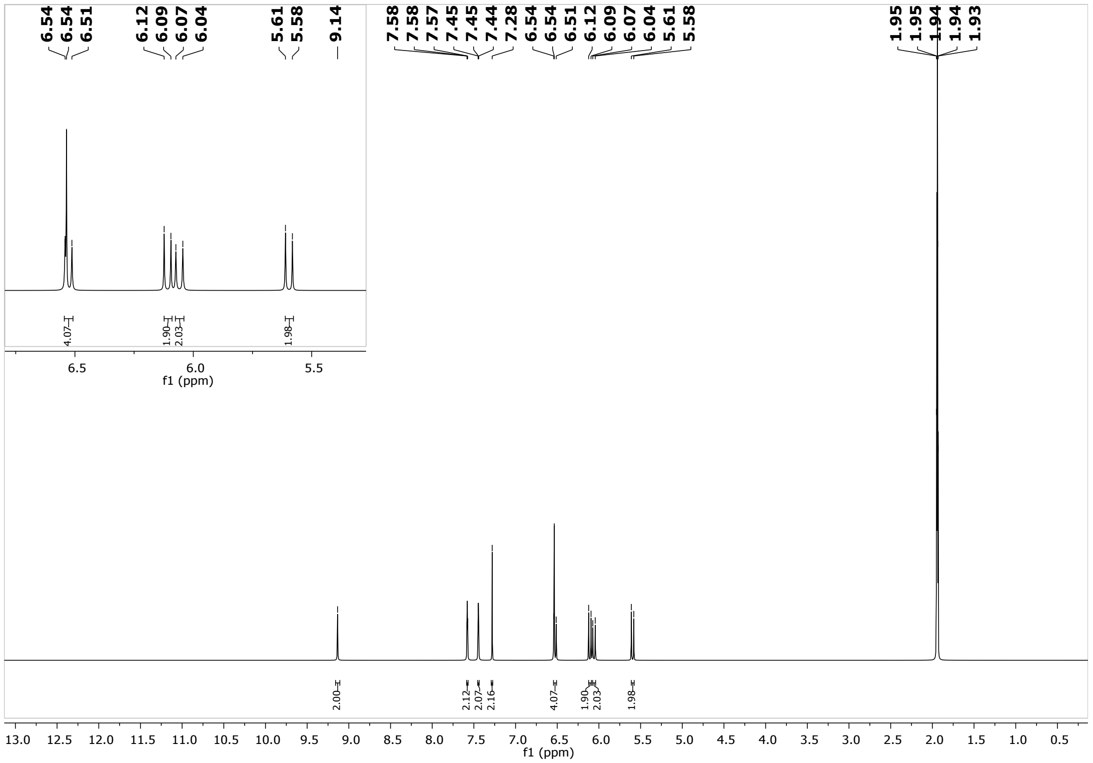

Fig. S18: Processed ^{1}H-NMR of **5** in MeCN-d_3.

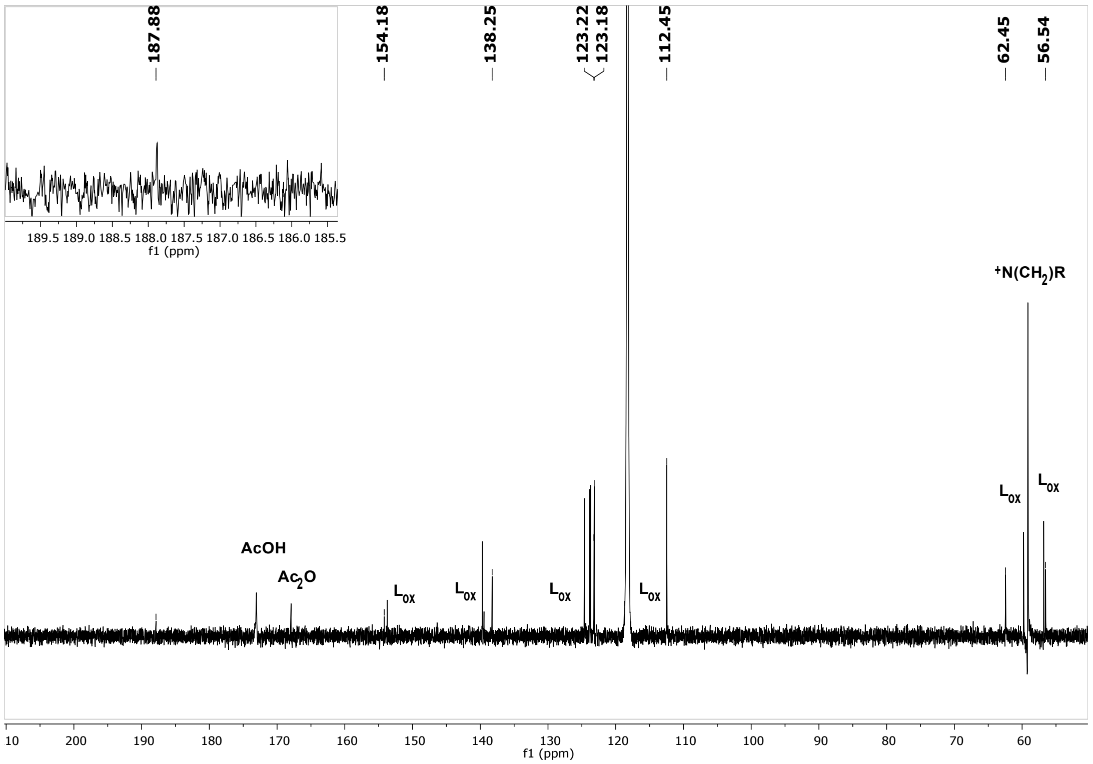

Fig. S19: Processed ^{13}C-NMR of **3-PF$_6$**, **5**, AcOH and [NBu$_4$][PF$_6$/OAc] in MeCN-d_3. All peaks marked with a chemical shift are assigned to **5**.

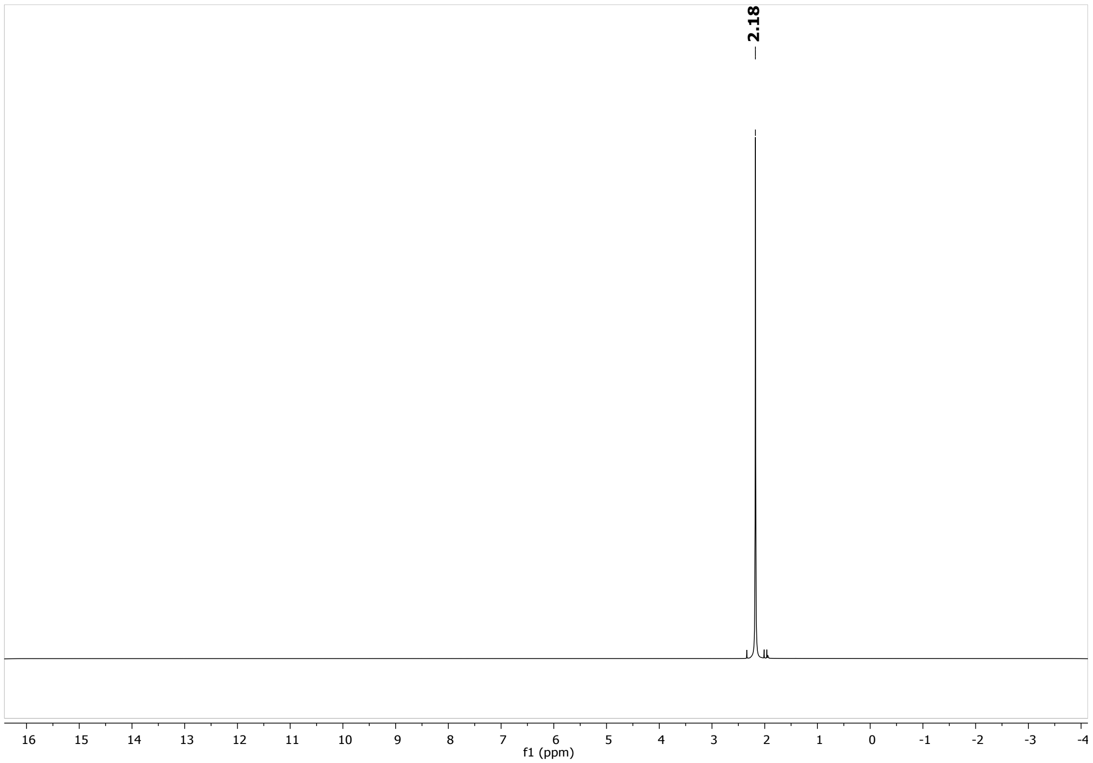

Fig. S20: Processed ^{1}H-NMR of **Ac$_2$O** in MeCN-d_3.

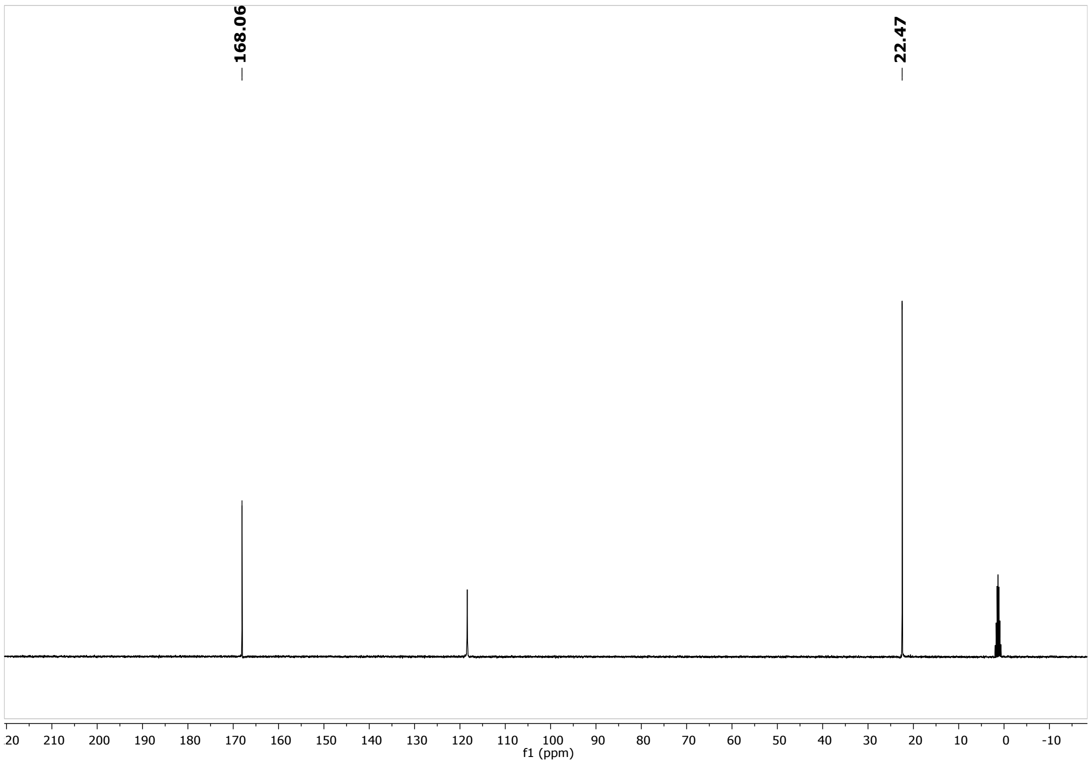

Fig. S21: Processed ^{13}C-NMR of **Ac_2O** in MeCN-d_3.

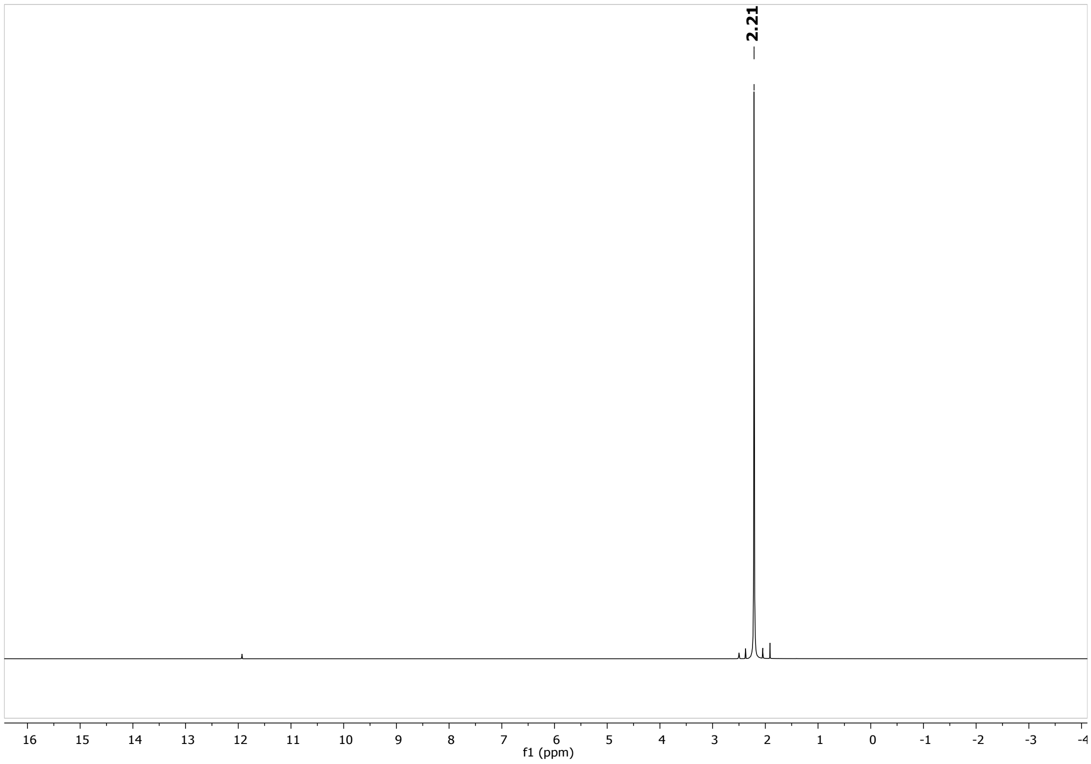

Fig. S22: Processed ^{1}H -NMR of **Ac_2O** in DMSO-d_6.

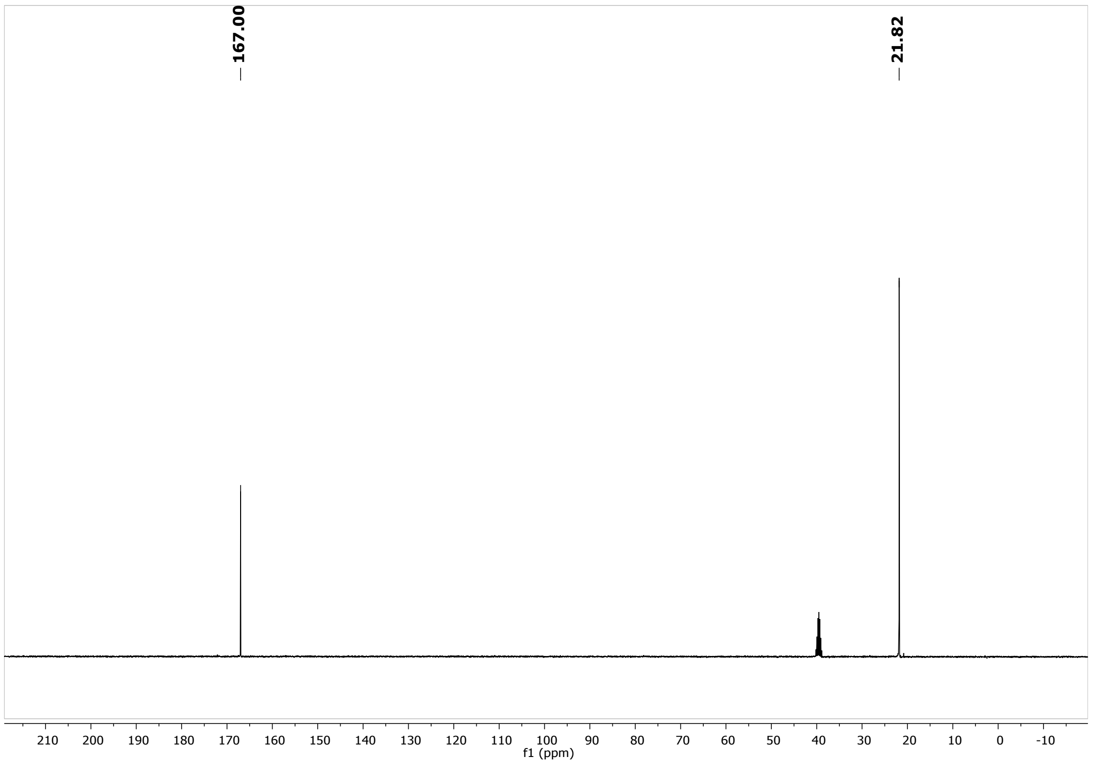

Fig. S23: Processed ^{13}C-NMR of **Ac_2O** in DMSO-d_6.

5. ^{1}H NMR Kinetics

5.1. ^{1}H NMR kinetic of the formation of 3 in DMSO-d_6.

A NMR-tube was charged with **H_4L-PF_6** (10 mg, 0.011 mmol) and it was dissolved with degassed DMSO-d_6 (0.4 mL). A blank NMR (t=0) was taken and successively Cu(II)$(OAc)_2$*H_2O (4.40 mg, 0.022 mmol) was added inside the Glovebox. The NMR-tube was well shaken and every 5 min. ^{1}H-spectra were collected at r.t. during the course of 60 min. During this time the color of the solution changed from blue to almost colorless. To complete the reaction from **H_4L-PF_6** to **1** the sample was heated at 40 °C for 10 min. and then stored at r.t. for 3 h. NBu_4OAc (7.2 mg, 0.022 mmol) was added to the now colorless, clear solution and another spectra was collected. After heating to 100°C for 1 min. the yellow solution was cooled to r.t. and a final spectrum of **3** was collected.

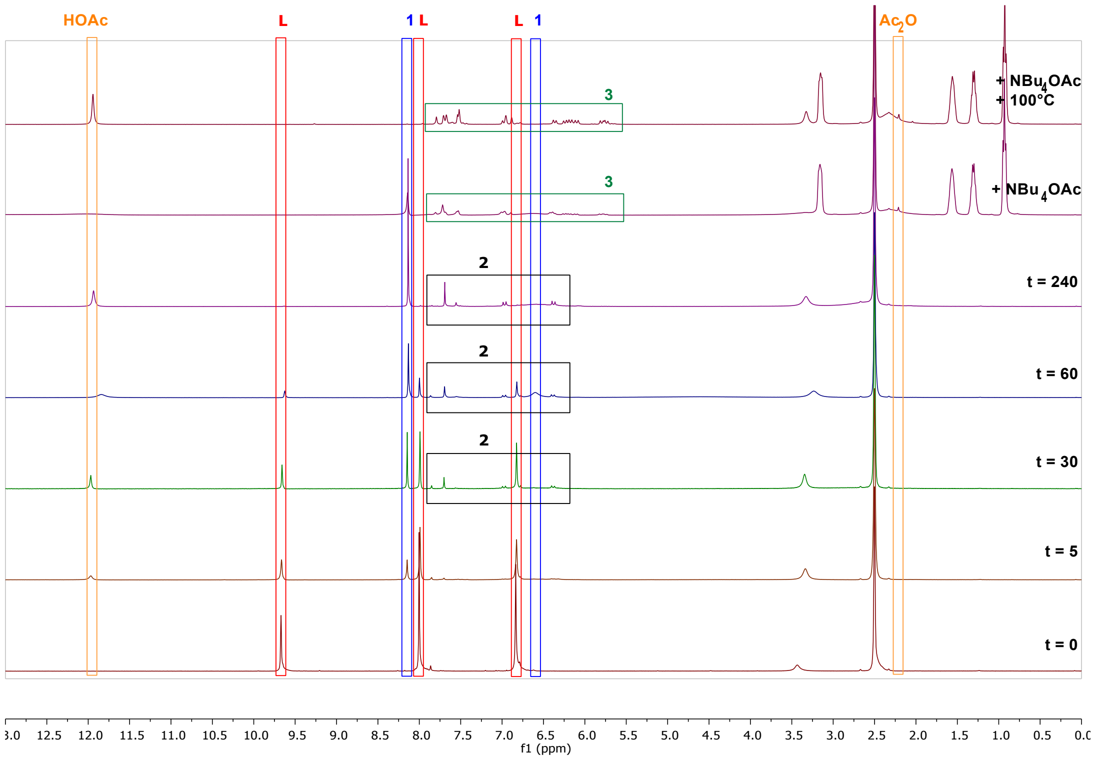

Fig. S24: Kinetic data of the formation of **3** in DMSO-d_6 monitored by ^{1}H-NMR.

5.2. ^{1}H NMR kinetic of the formation of 3 in MeCN-d_3.

A NMR-tube was charged with **H_4L-PF_6** (10 mg, 0.011 mmol) and it was dissolved with degassed MeCN-d_3 (0.4 mL). A blank NMR (t=0) was taken and successively Cu(II)(OAc)$_2$*H_2O (4.40 mg, 0.022 mmol) was added inside the Glovebox. The NMR-tube was well shaken and every 5 min. ^{1}H-spectra were collected at r.t. during the course of 180 min. at 50°C. During this time the color of the solution changed from blue to almost colorless. To complete the reaction from **H_4L-PF_6** to **4** and **5** the sample was heated at 80 °C for 10 min. and then stored at r.t. over night. NBu_4OAc (7.2 mg, 0.022 mmol) was added to the colorless solution at r.t., clear solution and after shaking another spectra was collected. After heating to 80°C for 5 min. the now yellow solution was cooled to r.t. and a final spectrum of **3** was collected.

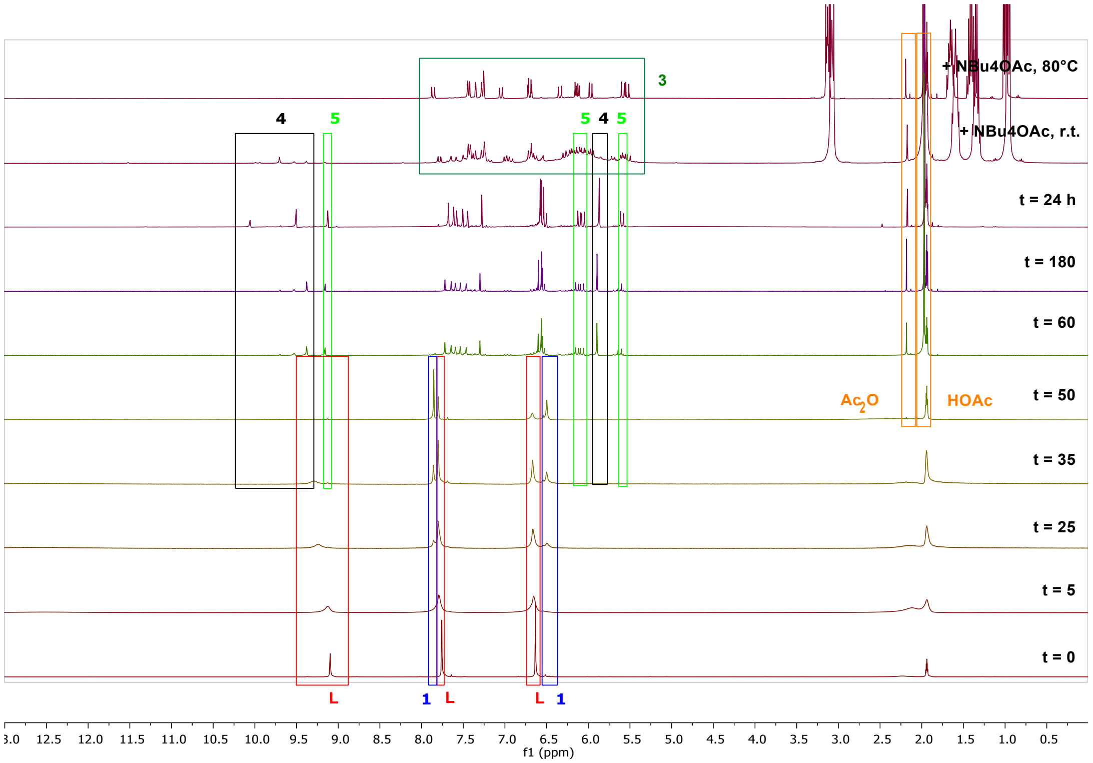

Fig. S25: Kinetic data of the formation of **3** in MeCN-d_3 monitored by ^{1}H-NMR.

5.3. ^{1}H NMR of 2 at on air in DMSO-d_6.

A NMR-tube was charged with **2** (10 mg, 0.012 mmol) and was dissolved with dry and degassed DMSO-d_6 (0.4 mL). A blank ^{1}H-NMR (t=0) was taken and during three weeks several ^{1}H NMR spectra were collected. Between the measurements, the solution was stored in an open NMR tube on air. After three weeks the NMR-tube was heated to 100°C for 30 min and the final NMR was collected, containing a mixture of **1** and **3**. The former colourless solution turned green. The ^{13}C-NMR after 21 d confirms the presence of **1** in the solution.

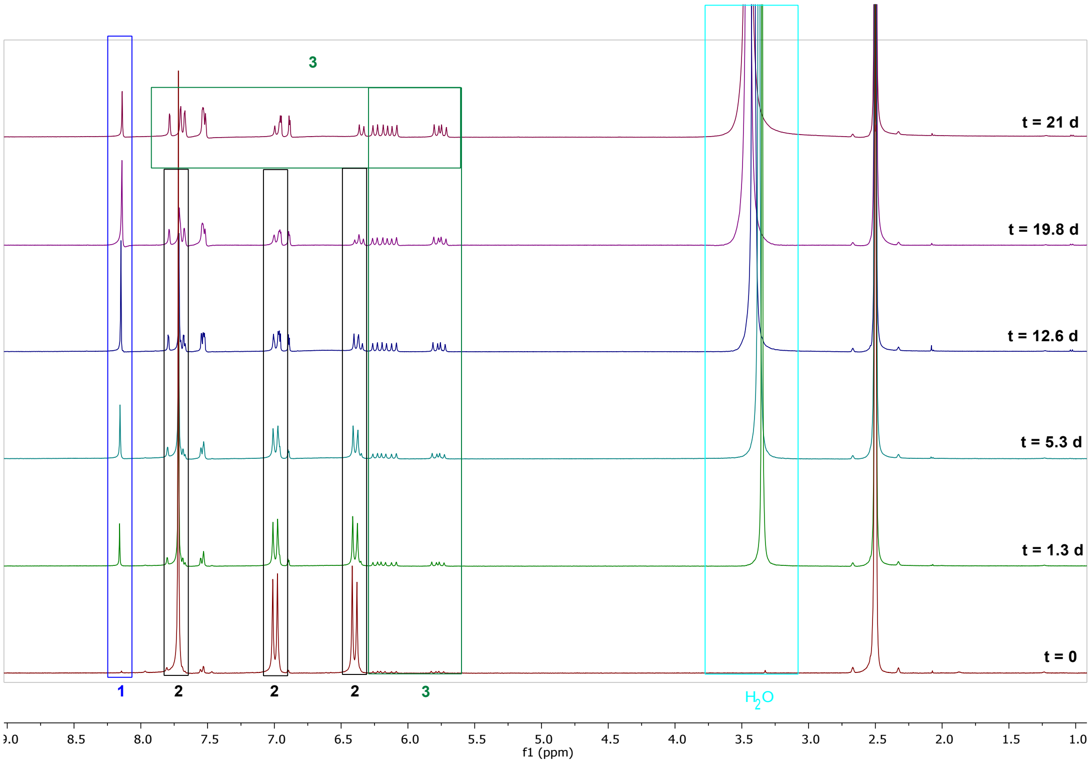

Fig. S26: Degradation of **2** forming **1** and **3** in DMSO-d_6 monitored by ^{1}H-NMR.

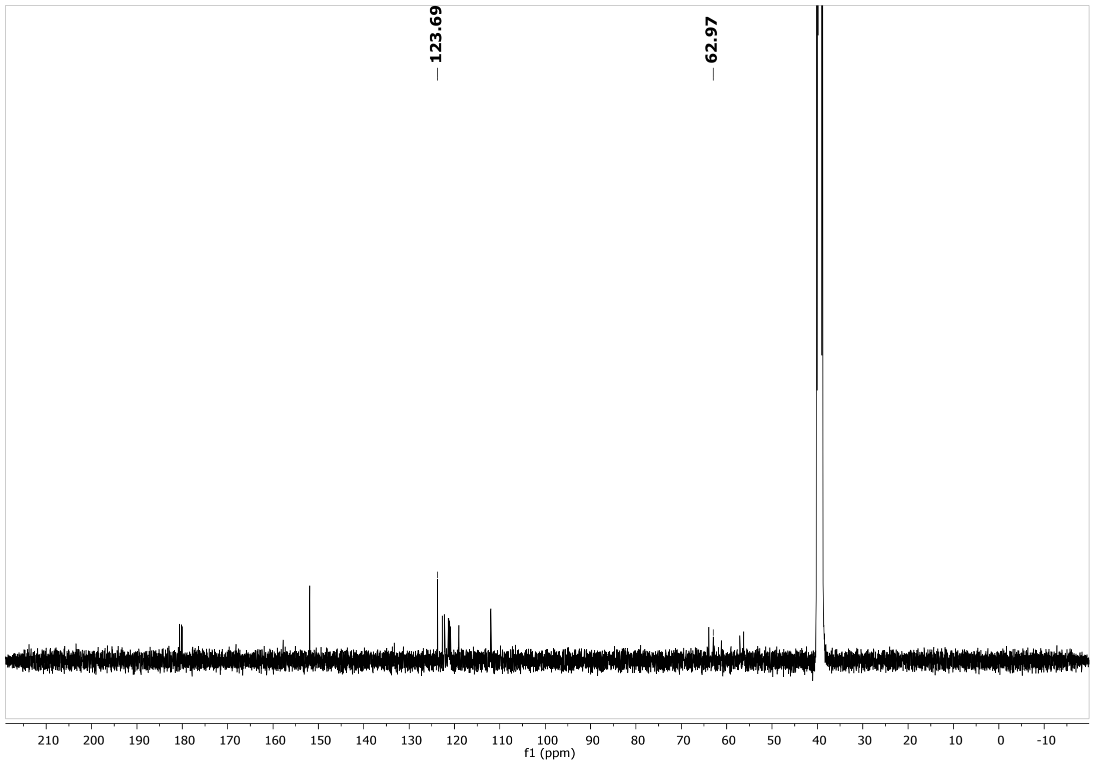

Fig. S27: Processed ^{13}C-NMR of **5.3** after 21 days in DMSO-d_6. The labelled peaks are assigned to **1**. All other peaks can be assigned to **3**.

5.4. ^{1}H NMR of 3 at various temperatures in MeCN-d_3.

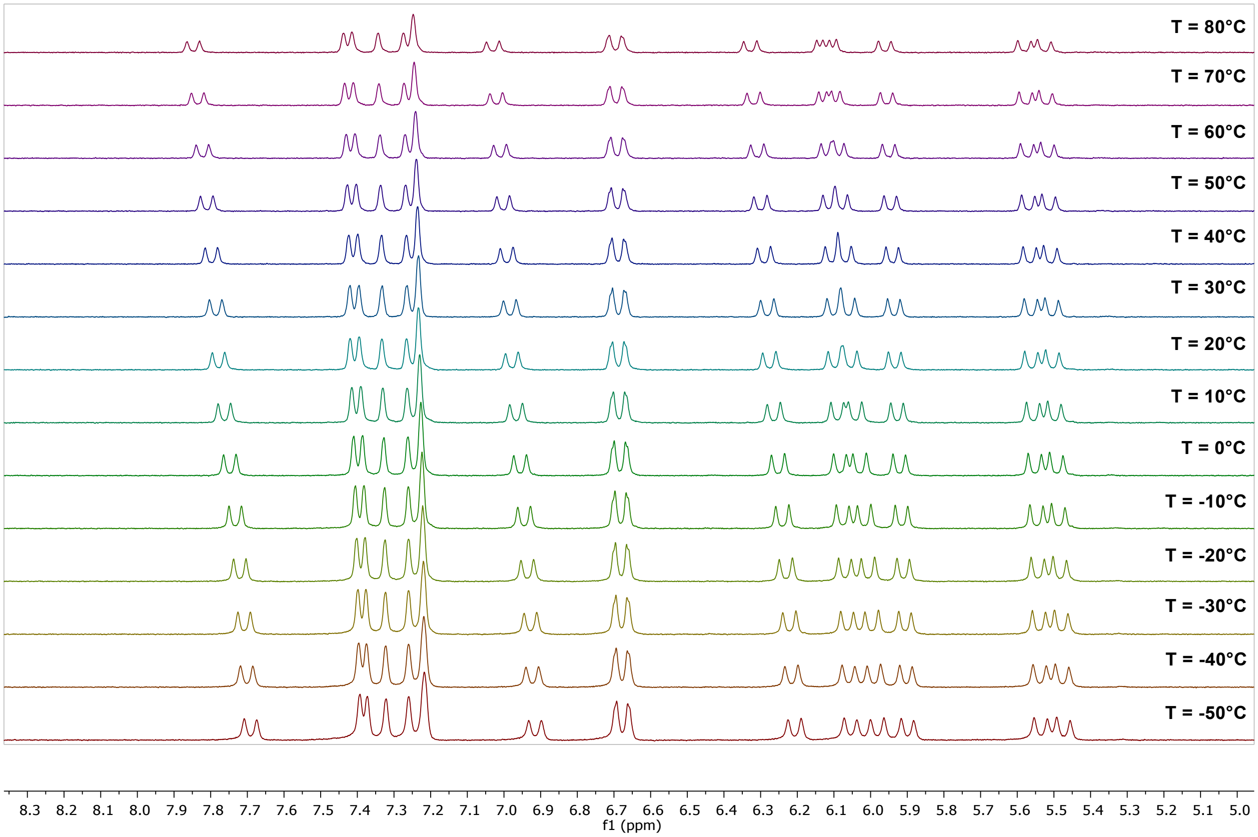

Fig. S28: VT-NMR of compound **3** in MeCN-d_3 in a temperature range from -50 to 80 °C.

5.5. ^{1}H NMR of 1 at various temperatures in MeCN-d_3.

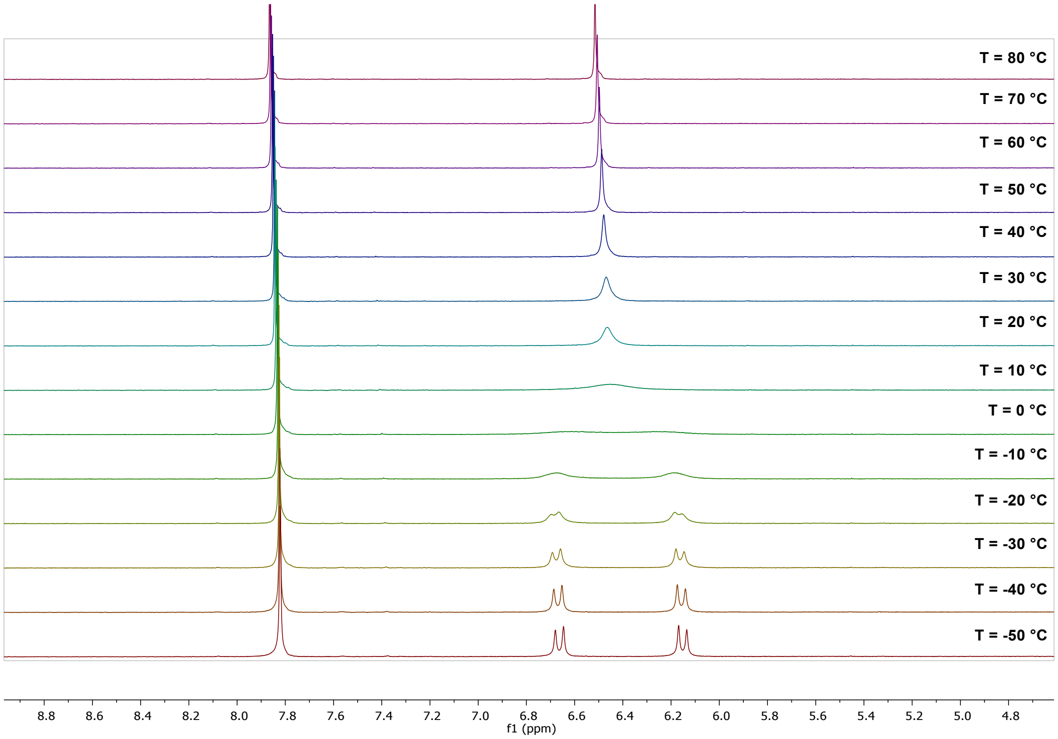

Fig. S29: VT-NMR of compound **3** in MeCN-d_3 in a temperature range from -50 to 80 °C.

5.6. ^{1}H NMR of 1 titrated with NBu_4OAc in DMSO-d_6 at r.t.

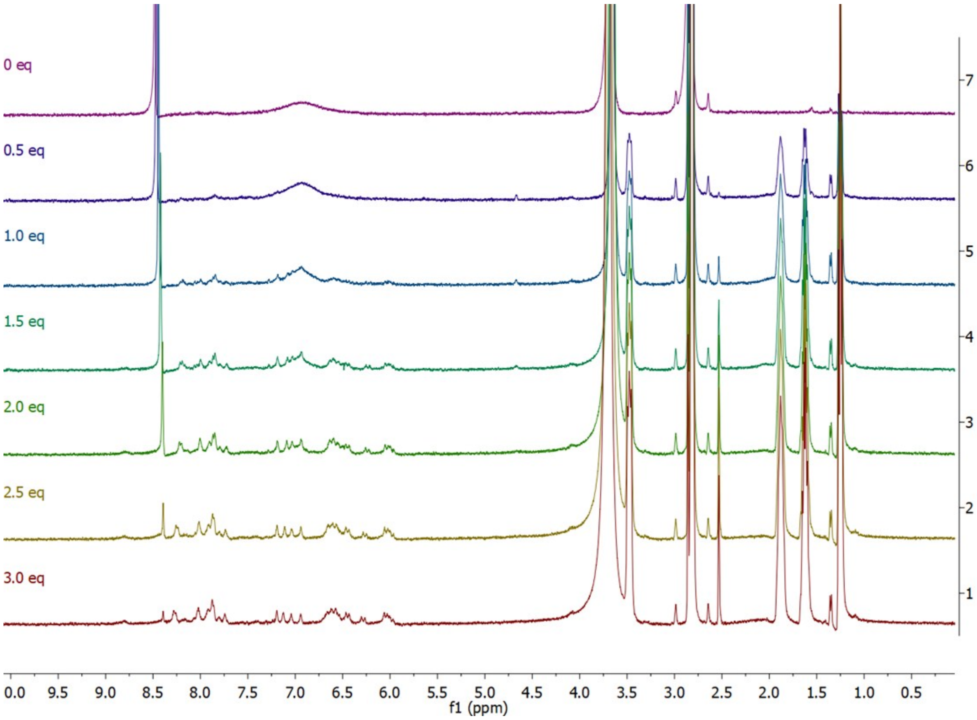

Fig. S30: ^{1}H-NMR titration of compound **1** in DMSO-d_6 with NBu_4OAc at room temperature.

6. Literature

1. *APEX suite of crystallographic software, APEX 2, version 2008.4.*, Bruker AXS Inc., Madison, Wisconsin, USA (2008).
2. *SAINT, version 7.56a, SADABS, version 2008.1*, Bruker AXS Inc., Madison, Wisconsin, USA (2008).
3. Hübschle, C. B.; Sheldrick, G. M.; Dittrich, B. *SHELXLE, J. Appl. Crystallogr.* **44**, 1281-1284 (2011).
4. Sheldrick, G. M. *SHELXL-2014*, University of Göttingen, Göttingen, Germany (2014).
5. Sheldrick, G. M. *SHELXL-97*, University of Göttingen, Göttingen, Germany (1998).
6. Wilson, A. J. C. *International Tables for Crystallography*, Dordrecht, The Netherlands, Kluwer Academic Publishers (1992).
7. A. L. Spek, *Acta Cryst. D*, **65**, 148-155 (2009).
8. H. Schmidbaur and A. Schier, *Angew. Chem. Int. Ed.*, 2015, **54**, 746.
9. D. T. Weiss, P. J. Altmann, S. Haslinger, C. Jandl, A. Pöthig, M. Cokoja and F. E. Kühn, *Dalton Trans.*, 2015, **44**, 18329.
10. X. Hu, I. Castro-Rodriguez, K. Olsen and K. Meyer, *Organometallics*, 2004, **23**, 755.
11. B. Raible and D. Kunz, *Z. Naturforsch. B*, 2016, **71**, 659.
12. M. Slivarichova, A. Ahmad, Y.-Y. Kuo, J. Nunn, M. F. Haddow, H. Othman and G. R. Owen, *Organometallics*, 2011, **30**, 4779.
13. V. G. Shtyrlin, N. Y. Serov, D. R. Islamov, A. L. Konkin, M. S. Bukharov, O. I. Gnezdilov, D. B. Krivolapov, O. N. Kataeva, G. A. Nazmutdinovae and F. Wendlere, *Dalton Trans.*, 2014, **43**, 799.
